# Beta-Catenin/HuR Post-Transcriptional Machinery Governs Cancer Stem Cell Features in Response to Hypoxia

**DOI:** 10.1371/journal.pone.0080742

**Published:** 2013-11-15

**Authors:** Gabriele D’Uva, Sara Bertoni, Mattia Lauriola, Sabrina De Carolis, Annalisa Pacilli, Laura D’Anello, Donatella Santini, Mario Taffurelli, Claudio Ceccarelli, Yosef Yarden, Lorenzo Montanaro, Massimiliano Bonafé, Gianluca Storci

**Affiliations:** 1 Department of Experimental, Diagnostic and Specialty Medicine, University of Bologna, Bologna, Italy; 2 Department of Biological Regulation, Weizmann Institute of Science, Rehovot, Israel; 3 Center for Applied Biomedical Research (CRBA) S. Orsola-Malpighi University Hospital, University of Bologna, Bologna, Italy; 4 Centro Interdipartimentale di Ricerche sul Cancro Giorgio Prodi-CIRC, University of Bologna, Bologna, Italy; 5 Clinical and Surgical Science, University of Bologna, Bologna, Italy; University of Alabama at Birmingham, United States of America

## Abstract

Hypoxia has been long-time acknowledged as major cancer-promoting microenvironment. In such an energy-restrictive condition, post-transcriptional mechanisms gain importance over the energy-expensive gene transcription machinery. Here we show that the onset of hypoxia-induced cancer stem cell features requires the beta-catenin-dependent post-transcriptional up-regulation of CA9 and SNAI2 gene expression. In response to hypoxia, beta-catenin moves from the plasma membrane to the cytoplasm where it binds and stabilizes SNAI2 and CA9 mRNAs, in cooperation with the mRNA stabilizing protein HuR. We also provide evidence that the post-transcriptional activity of cytoplasmic beta-catenin operates under normoxia in basal-like/triple-negative breast cancer cells, where the beta-catenin knockdown suppresses the stem cell phenotype *in vitro* and tumor growth *in vivo*. In such cells, we unravel the generalized involvement of the beta-catenin-driven machinery in the stabilization of EGF-induced mRNAs, including the cancer stem cell regulator IL6. Our study highlights the crucial role of post-transcriptional mechanisms in the maintenance/acquisition of cancer stem cell features and suggests that the hindrance of cytoplasmic beta-catenin function may represent an unprecedented strategy for targeting breast cancer stem/basal-like cells.

## Introduction

Stem cells are harbored in specialized niches where low oxygen tension (hypoxia) contributes to the regulation of self-renewal and differentiation [1–3]. In fact, hypoxia maintains the undifferentiated state of embryonic, hematopoietic, mesenchymal and neural stem/progenitor cells [2]. Hypoxia in tumors is associated with poor prognosis [4]. Cells in hypoxic tumor regions stabilize hypoxia-inducible-factors (HIFs) and activate adaptive gene expression leading to cancer aggressiveness through cell survival and dedifferentiation [1,5]. Moreover, HIF activity in a rare subset of hypoxic tumor cells confers stem cell-like properties [1–3].

Beta-catenin is a crucial regulator of normal and cancer stem cell self-renewal [6,7]. In response to various stimuli, beta-catenin induces the expression of target genes by shuttling into the nucleus, where it interacts with TCF/LEF family transcription factors [6]. 

Physical interaction between HIF-1alpha, the major player in hypoxia response, and beta-catenin has been described [8]. Moreover, beta-catenin and HIF-1alpha synergistically facilitate hypoxia survival in colon cancer cells and self-renewal in neural stem cells [8,9].

 Hypoxia is an energy restrictive condition, which markedly decreases total *de novo* transcription and promotes the energy-saving post-transcriptional regulation of pre-existing mRNAs [10–12]. Recent studies report that beta-catenin modulates the half-life of cytoplasmic mRNAs [13–17]. These data lead to us surmise that the post-transcriptional activity of beta-catenin plays an important role in the adaptation of cancer cells to hypoxia. Here we analyzed the role of beta-catenin in the mRNAs production and stabilization of two important breast cancer stem cell regulatory genes, i.e. carbonic anhydrase 9 (CA9) and SNAI2. The expression of CA9 and SNAI2 genes is induced by hypoxia, via HIF1-alpha-mediated transcriptional up-regulation [18–20]. CA9 expression regulates pH in the hypoxic microenvironment to promote survival and proliferation of cancer stem cells [21,22]. Therefore CA9 has been suggested as an anticancer therapy target [23,24]. SNAI2, also known as SLUG, is an important functional suppressor of human breast progenitor cell lineage commitment and differentiation, promoting normal and tumor mammary gland stem/progenitor cells state [25,26]. 

We here report that the cytoplasmic accumulation of beta-catenin in response to hypoxia activates a post-transcriptional de-differentiation and survival program, which enhances stem cell features in breast cancer cells. The phenomenon relies upon the ability of cytoplasmic beta-catenin to bind and stabilize SNAI2 and CA9 mRNAs. We also provide evidence that the post-transcriptional activity of cytoplasmic beta-catenin operates under normoxia in basal-like/triple-negative breast cancer cells. The basal-like/triple-negative breast cancer is a poorly differentiated and aggressive breast cancer subtype, characterized by the expression of a stem cell-like gene profile [27,28], by the cytoplasmic localization of beta-catenin [29–31] and by CA9 and SNAI2 gene overexpression [32,33]. In such cells, beta-catenin knockdown dramatically diminished the stability and expression of CA9 and SNAI2 mRNAs and blunts the stem cell phenotype *in vitro* and the xenograft-establishing capability *in vivo*. Moreover, beta-catenin beta-catenin was able to regulate the mRNA stability of several EGF-induced mRNAs, among which the pro-inflammatory cytokine Interleukin 6 (IL6), an acknowledged cancer stem growth factor [21,34]. The data here reported highlight the role of post-transcriptional mechanisms in the regulation of cancer stem cell features, and identify beta-catenin as a pivotal post-transcriptional player in the breast cancer stem cell phenotype. We propose that the hindrance of beta-catenin post-transcriptional activity here described represents a not yet explored strategy to target breast cancer stem/basal-like cells.

## Materials and Methods

### Cell lines, chemicals and hypoxia exposure

We used MCF7 cells as a model of well-differentiated luminal breast carcinoma and MDA-MB-468 and MDA-MB-231 cells as a model of poorly differentiated basal-like breast carcinoma [35]. All the cell lines were purchased from American Type Culture Collection (ATCC, Manassas, VA, USA). MCF7, MDA-MB-468 and MDA-MB-231 were grown in RPMI-1640, DMEM/F12 and DMEM respectively. All media were supplemented with 10% FBS, 1% of penicillin and streptomycin and 1% of glutamine Euroclone (Milan-Italy). All the normoxic cell cultures were kept at 37°C in a 5% CO_2_-humidified atmosphere. Hypoxia (1%pO_2_, 5%pCO_2_, 94%pN_2_) was obtained in an *invivo*300 hypoxia cabinet (Ruskinn Technology, Bridgend, UK) for 48 h. Cell death was evaluated by Trypan blue staining.

### Generation of MS from primary breast cancer tissues and cell lines

MS from human mammary gland tissues were obtained as previously described [21]. Briefly, tissue specimens were placed in sterile Epicult media (StemCell Technologies, Vancouver, Canada), minced with sterile scalpels, and incubated for 6–12 hours in presence of 1,000 U Collagenase/Hyaluronidase enzyme mix (StemCell Technologies) and filtered through a 40 μm nylon mesh (Becton Dickinson), re-suspended in complete MEGM and plated into 1cm^2^ low attachment plates. Secondary MS generation was obtained by incubating primary or consecutive MS in 1× Trypsin-EDTA solution (Cambrex) for 3 minutes, filtration throughout a 40-μm nylon mesh and single cell re-suspension in complete MEGM. MS were scored at day 7. Clearance was obtained from the S.Orsola-Malpighi Hospital ethical committee, University of Bologna (Prot. n. 75/2011 to LM and MB and MT). Written informed consent was obtained from patients whose tissues were used in the study. MCF7-MS were generated by seeding 5000 MCF7 cells in low attachment 24 well and scored at day 5.

### Stable Beta-catenin and SNAI2 knockdown in MCF7, MDA-MB-468 and MDA-MB-231 cells

Stable beta-catenin knockdown in MCF7, MDA-MB-468 and MDA-MB-231 cells was achieved by transducing the pCtoGMB-GFP retroviral vector carrying human beta-catenin specific 19nt coding sequence (GAGCCTCTATACCACCCAC) by a PCR-based cloning strategy, as previously described for shSNAI2 vector [20]. shBeta and shSNAI2-infected cells were selected by GFP cytofluorimetric sorting, as previously described [20]. 

### Immunofluorescence and Confocal microscopy analysis

Cultured cells were seeded onto glass coverslips at 60% confluence, while MS were cultured in BD reduced Matrigel^TM^ (Becton Dickinson, Franklin Lakes, NJ). Cells were fixed with 2% paraformaldehyde and permeabilized with 0.2% Triton X-100. Cells were incubated with anti-beta-catenin primary antibody (clone E5, Santa Cruz Biotechnologies, Santa Cruz, CA) and secondary anti-mouse FITC conjugated antibody (Dako Cytomation, Glostrup, DK). Nuclei were counter-stained with propidium iodide and mounted in the anti-fade Pro-long reagent mounting medium (Molecular Probes Inc, Eugene, Oregon, USA). Images were captured using a Zeiss LSM 710 on a Zeiss Observer Z1 or a Leica DMI 6000B inverted microscope (Leica Microsystems GmbH, Wetzlar, Germany). 

### Transient transfection of siRNA and expression vectors

CA9-, HuR-, and SNAI2-specific siRNAs and non-specific siRNA control (siSCR) oligonucleotides (Stealth^TM^ technology) with a matched GC content were purchased from Invitrogen (Carlsbad, CA, USA). Plasmids encoding wild type beta-catenin (Beta-WT; pCI-neo), dominant negative pcDNA4-TCF4DN (TCF4DN), and pTER+shBeta-catenin (shBeta) encoding vector were obtained from Dr. Marc Van De Wetering (Hubrecht Institute, Utrecht, Netherlands) and Bert Vogelstein (Johns Hopkins University, MD, USA). Plasmid encoding HIF1-alpha was obtained from Eric Huang (Department of Neurosurgery, University of Utah, Salt Lake City, Utah). Wild type EGFR encoding vector was obtained from Pier Paolo di Fiore (European Institute of Oncology, Milan, Italy); siRNAs or plasmids were transfected to MCF7 cells (10^5^ cells in a 3 cm^2^ well) at a concentration of 1 µg/well for 72h or 24 h respectively using lipofectamine 2000 (Life Technologies, Gran Island, NY), or Jet-Pei (Polyplus, Illkirch, France) in the case of MS. MCF7 cells stably transduced with a retroviral vector encoding a p53 dominant inactivating mini-protein (p53D) were previously described [36].

### Gene promoter and mRNA 3’UTR luciferase reporter assays

Carbonic anhydrase-9 luciferase plasmid (CA9-Luc, spanning the -170 to +34 region of CA9 promoter), was kindly provided by Dott. Jaromir Pastorek; HIF1alpha responding luciferase plasmid, HRE-Luc, was kindly provided by Dr. Giovanni Melillo (Tumor hypoxia laboratory, National Cancer Institute, Frederick, MD, USA). SNAI2-Luc plasmid was kindly provided by Dr. Togo Ikuta (Saitama Cancer Centre, Saitama, Japan); TopFlash, was a gift of Dr. Rolf Kemler (Max Planck Institute, Heidelberg, Germany); Estrogen Response Element (ERE-Luc) reporter was provided by Rakesh Kumar (Department of Molecular and Cellular Oncology, MD Anderson Cancer Center, Houston, Texas); Thymidine Kinase Renilla luciferase reporter plasmid (Promega, Madison, WI) was used as control in luciferase assay after 24 hours using the Dual-Luciferase® Reporter Assay System (Promega), according to the manufacturer’s instructions. Data are expressed as fold changes of firefly over renilla luciferase activity. CA9 and SNAI2 3’UTR luciferase assay constructs were obtained from Genecopoeia (Rockwell, Maryland, USA). Each cell line was transfected with 3’UTR-CA9/SNAI2 vector or with control pEZX-MT01 empty vector. Data are presented as ratio of 3’UTR-CA9/SNAI2 over control vector, according to manufacturer instructions.

### RNA extraction and cDNA amplification

Total RNA was extracted from cells using TRIzol® Reagent according to the manufacturer’s protocol (Invitrogen, Carlsbad, CA). Primers and PCR conditions are reported in Table S1.

### Real-Time PCR analysis

Real-Time PCR analysis was performed by TaqMan approach in aiCycler iQ™ Real-Time PCR DetectionSystem (Applied Biosystems, Carlsbad, CA, USA). Each sample was analyzed in triplicate. Sets of primers and fluorogenic probes specific for CA9, SNAI2, ESR1, IL6 and CD44 mRNAs were purchased from Applied Biosystems. The reactions were incubated at 50° for 2 min; 95°C for 15 min followed by 45 cycles of 95°C for 15 s and 60°C for 1 min. The relative amount of the target mRNA was calculated equal to 2 ^− (Δ*C*t target mRNA- Δ*C*t control)^, using human beta-glucuronidase mRNA as control, except for mRNA immunoprecipitation assay, mRNA stability assay, and cytoplasmic fractionation assay.

### High Throughput Gene Expression Measurement with Real Time PCR in a Microfluidic Dynamic Array (Fluidigm® Real-Time PCR)

RNA was isolated using the PerfectPure RNA Cultured Cell Kit with DNAse-1 digestion (5 Prime, Hamburg, Germany). cDNA was synthesized using the SuperScriptII first-strand synthesis kit (Invitrogen, Carlsbad, CA). For qPCR of pre-mRNA and mRNA, respectively, forward primers were positioned in the second intron and exon, and a shared reverse primer (here called universal) was positioned in the third constitutive exon. For genes in which universal primers sequences were not available, 2 couples of primers were designed in order to amplify respectively intronic and exonic sequences. Each cDNA sample was mixed with the pool of primers for a pre-amplification reaction of 14 cycles with Reagent (Fluidigm PN 85000735) and TaqMan PreAmp Master Mix, according to the manufacturer’s instructions. The Preamplified cDNA was diluted 1:100. The modified 2x TaqMan universal Master Mix was added to the diluted cDNA in order to obtain a final concentration of Master Mix 1x in the samples. The chip was primed in the NanoFlexTM 4-IFC Controller prior to loading the samples and assay reagents into the inlets. Data were analyzed by using Ct values and ΔΔCt values. Each sample was in triplicates and normalized either on GAPDH, B2M and TBP. Primer sequences are listed in Table S2.

### Western Blot and co-immunoprecipitation assay

Total proteins were extracted with RIPA Buffer (25mM Tris-HCl, pH 7.6, 145 mM NaCl, 1% NP-40, SDS 0.1%, Na-Deoxycolate 0.5%) added with Protease Inhibitor Cocktail (Roche, Basel, Switzerland). Western blot analysis was performed using the following antibodies: Laminin, Beta-tubulin, anti-beta-catenin (E5), anti-Actin (C4) purchased from Santa Cruz Biotechnology, (Santa Cruz, CA); anti-HuR (Molecular probes, Invitrogen) purchased from molecular probes; anti-CA9 (AF2188) purchased from R&D (Minneapolis, MN) and clone M75 kindly provided by Prof J. Pastorek (Slovak academy of Science (Bratislava, Czech Republic). Co-immunoprecipitation assay was performed in CO-IP buffer (50mM tris-HCl pH 7.5, 150 mM NaCl, 5mM EDTA, 1% NP40) added with protease inhibitors (Roche).

### mRNA immunoprecipitation assay

mRNA immunoprecipitation assay was performed following standard protocol which preserves mRNA/protein interaction using the Polysome lysis buffer [37]. In brief, cultured cells were suspended in lysis buffer (100mM KCl, 5mM MgCl_2_, 10mM Hepes, pH 7.0, 0.5% Nonidet P-40) supplemented with RNase and protease inhibitors. Proteins were immunoprecipitated using protein A beads (Santa Cruz) and either anti-Beta-catenin (E5, Santa Cruz) or anti-HuR (Molecular probes), or normal IgG (sc-2025, Santa Cruz) mouse antibodies, in NT-2 buffer (50mM Tris, pH 7.4, 150mM NaCl, 1mM MgCl_2_, 0.05% Nonidet P-40), supplemented with RNAase inhibitor (40U/ul), DTT (1mM). Immunoprecipitates were lysed in Trizol® reagent (Life technologies). cDNAs were analyzed by RT-PCR (MCF7 cells, MDA-MB-468 and MDA-MB-231 cells) or by Real Time PCR (T-MS). Data are presented as fold increase of each mRNA bound to the specific antibody over the control IgG [37]. 

### Actinomycin D mRNA stability assay

Stability assay for mRNA was performed by exposing cells to Actinomycin D at 100ng/ml and assessing level of each specific mRNA at different time points (0 to 6 h): mRNA level at first hour after Actinomycin exposure was taken as reference point (time 0).

### Cytoplasmic pre-ribosome and 40S ribosome fractionation procedure

For isolations of ribosomal fractions two 10-cm plates of 80% confluent cells were lysed in lysis buffer (10 mM NaCl, 3 mM MgCl2, 20 mM Tris-HCl pH 7.5). Organelle-free cytoplasm was obtained by saving supernatants after centrifugation at 14000 × g for 5 min at 4°C. Ribosomal fractions were then separated by centrifugation of cytoplasmic lysate at 100.000 g (36.000 rpm) in a SW41Ti rotor (Beckman) onto a 15-50% sucrose gradient added with RNase inhibitor and cycloheximide. Fractions corresponding to 40S ribosome subunit or low-density pre-ribosomal cytoplasm were used for RNA and protein extraction (Material S1A-B). RNA extraction was performed by TRI-Reagent (Ambion). Proteins were extracted with TCA at 4°C, dried at 95°C, and re-suspended in 1X Laemli buffer. mRNA were assessed by Real Time PCR. Data are presented as fold increase of each mRNA in beta-catenin knockdown cells over controls.

### Cytofluorimetric analysis

Cells were washed once with phosphate-buffered saline (PBS) and then harvested with Cell Dissociation Solution Non-enzymatic 1x (Sigma). Detached cells were washed with PBS containing 5% FCS and 0.1% sodium azide (wash buffer) and re-suspended at the concentration of 0.5*10^6^ cells/100μl of wash buffer. Combinations of fluorochrome-conjugated monoclonal antibodies obtained from BD Bioscience Pharmigen (San Diego, CA, USA) against human CD44 (G44-26, APC; cat. #560890) and CD24 (PE-cy7; cat. # 561646) or their respective isotype controls were added to the cell suspension at concentrations recommended by the manufacturer and incubated at 4°C in the dark for 30 min. The labeled cells were washed in the wash buffer and then analyzed on a LSR II Flow Cytometer (BD Biosciences).

### Foci-forming assay

To test the ability of selected cell lines to form foci, cells were plated into six-well-plates, maintained at conﬂuence, replacing the medium each 3-4 days. Foci were scored at day 14^th^.

### Xenograft assay and tissue immunohistochemistry

2*10^6^ MDA-M6B-468 cells were injected in the mammary fat-pad of female nude mice. Tumor growth was monitored weekly and then removed after 10 weeks (Weizmann Institute Animal Care and Use Committee approved the animal experiment, IACUC n. 01990412-2). 1*10^6^ MDA-MB-231 cells were subcutaneously injected in the flank of nude mice. Tumor growth was monitored weekly and then removed after 4 weeks. (Animal Ethical Committee of University of Bologna approved the animal experiment, prot. n. 8134-X/10). Formalin-Fixed/Paraffine embedded tissues were stained with Hematoxylin-Eosin, and assessed by immunonistochemistry, with the following antibodies: anti-ESR1 (clone ID5, DakoCytomation, Glostrub, Denmark); anti-CDH1 (clone NCH38, Dako Cytomation), anti-SNAI2 (L40C6, Cell Signalling, Beverly, MA), anti-CA9 (clone M-75, kindly provided by J. Pastorek, Slovak Academy of Sciences, Bratislava).

### Statistics and bioinformatics

Bioinformatics analysis on AU-Rich element-containing mRNA was performed consulting the online database AREsite [38] (http://rna.tbi.univie.ac.at/cgi-bin/AREsite.cgi). Statistical analysis was performed using SPSS software. Data are presented as mean +/- s.d.; p values referring to *t* test are reported, unless otherwise specified (n=3).

## Results

### Hypoxia elicits breast cancer cell dedifferentiation and survival/proliferation by triggering CA9 and SNAI2 expression

**Figure 1:**
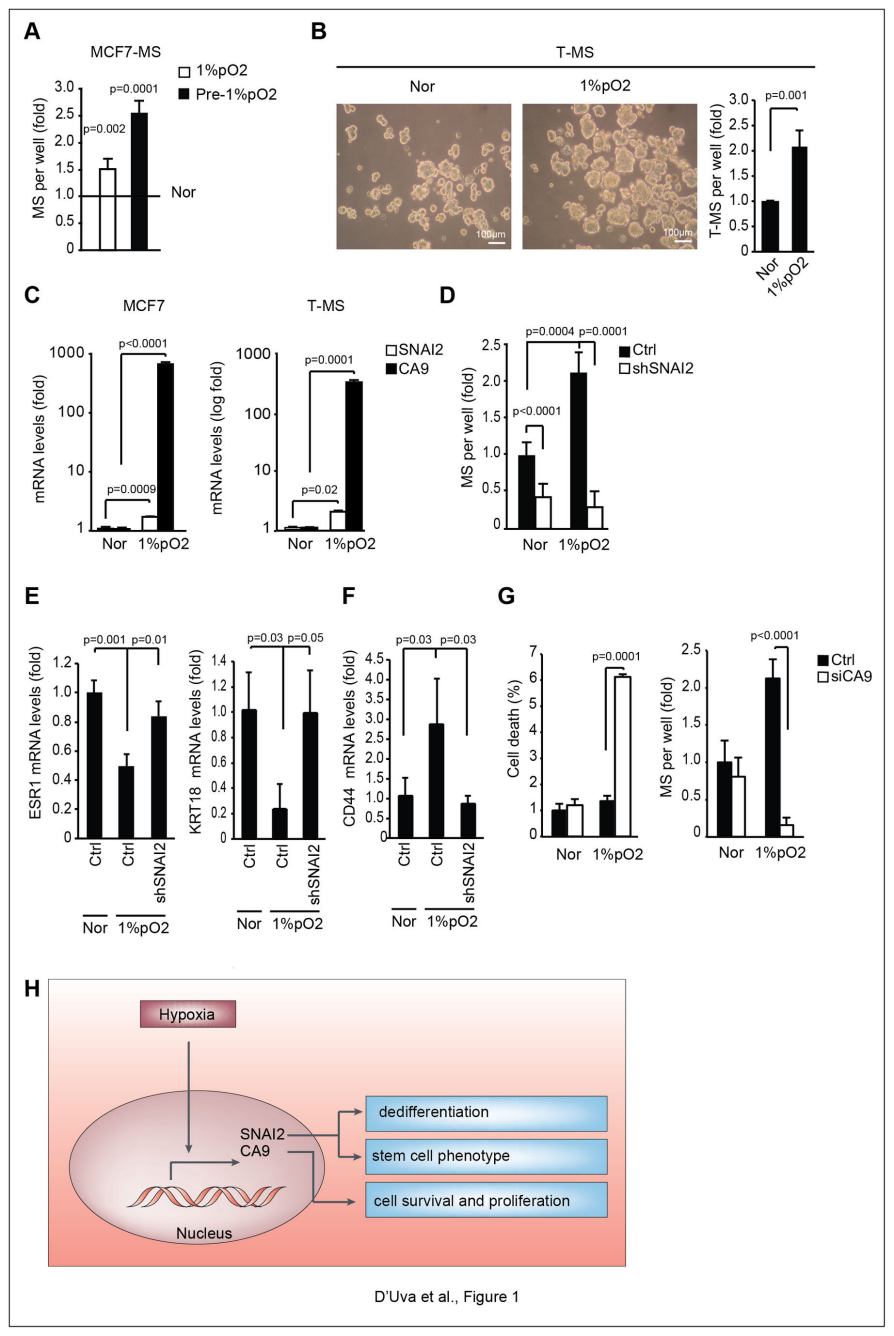
Hypoxia exposure induces breast cancer cell dedifferentiation and survival by triggering CA9 and SNAI2 expression. **A**, Mammosphere (MS) formation assay in MCF7 cells in response to hypoxia exposure (1%pO_2_) or after pre-exposure of adherent cells to 1%pO_2_ (Pre-1%pO_2_); MS forming capability of MCF7 cells in normoxia (Nor) is reported as basal value; **B**, Tumor MS formation assay (T-MS) in Nor/1%pO_2_ ductal breast carcinoma tissues-derived cells, n=5; representative pictures included; **C**, CA9 and SNAI2 mRNA in Nor/1%pO_2_ MCF7 cells and T-MS; **D**, MS formation assay of Ctrl/SNAI2-specific shRNA retroviral vector (shSNAI2)-transfected MCF7 cells exposed to Nor/1%pO_2_; **E**, Real-Time PCR analysis of ESR1 and KRT18 mRNA levels in Ctrl/shSNAI2 MCF7 cells exposed to Nor/1%pO_2_; **F**, Real-Time PCR analysis of CD44 mRNA levels in Ctrl/shSNAI2 MCF7 cells exposed to Nor/1%pO_2_; **G**, Cell death and MS assay in scramble (Ctrl) and CA9 siRNA (siCA9) transfected MCF7 cells exposed to Nor/1%pO_2_; **H**, Schematic representation of the role of CA9 and SNAI2 in the regulation of cancer stem cell features in response to the hypoxic microenvironment. Data are presented as mean +/- s.d.; p values refers to t test. n=3, unless otherwise specified.

*In vitro*, breast cancer stem/progenitor features are over-represented in mammospheres (MS) [39]. Our investigation was prompted by the observation that exposure or pre-exposure to hypoxia (1%pO_2_) increased the MS forming ability of MCF7 cells (Figure 1A), and of ductal breast carcinoma tissues-derived cells (T-MS, Figure 1B). We then observed that in MCF7 cells, as well as in T-MS, hypoxia increased the mRNA expression of two crucial breast cancer stem cell regulatory genes, namely carbonic anhydrase 9 (CA9) and SNAI2 (Figure 1C), via *de novo* mRNA production (Figure S1A). Importantly, SNAI2 shRNA knockdown reduced normoxic MS forming capability, as well as blunted hypoxia MS expansion (Figure 1D). Consistently, siRNA-mediated SNAI2 knockdown tampered hypoxic T-MS formation (Figure S1B). Moreover, shRNA-mediated SNAI2 knockdown halted the hypoxia-induced down-regulation of the epithelial differentiation markers estrogen receptor alpha (ESR1), keratin 18 (KRT18) and e-cadherin (CDH1) (Figure 1E and Figure S1C) and the hypoxia-induced up-regulation of CD44 expression (Figure 1F), a marker of breast cancer stem/progenitor cells [21,40]. Finally, in line with the pro-survival/proliferative role of CA9 [21,22], siRNA-mediated CA9 silencing increased cell death and hindered MS formation in hypoxic MCF7 cells (Figure 1G). These data show that hypoxia induces a SNAI2-dependent de-differentiation program and a CA9-dependent survival/proliferation program, leading to an increase in the stem/progenitor cells sub-population (Figure 1H).

### Beta-catenin increases the breast cancer stem cell phenotype in response to hypoxia independently of its nuclear transcriptional activity

**Figure 2:**
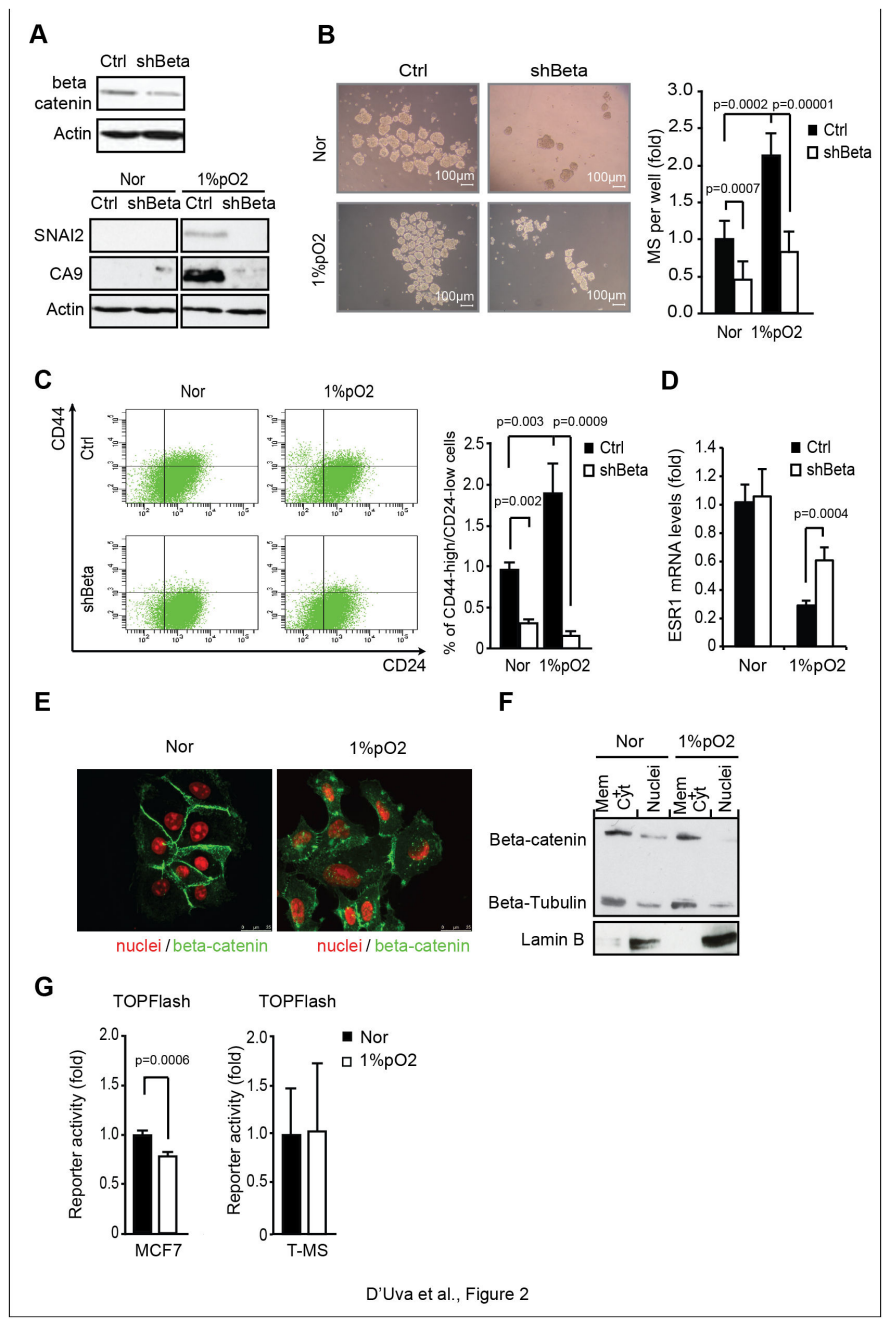
Beta-catenin enhances the breast cancer stem cell phenotype in response to hypoxia independently of its nuclear transcriptional activity. **A**, Western analysis (WB) of beta-catenin, SNAI2 and CA9 protein levels in Ctrl/shBeta MCF7 cells upon Nor/1%pO_2_ conditions; **B**, MS forming assay in stable beta-catenin silenced (shBeta) MCF7 cells upon Nor/1%pO_2_ conditions; **C**, Cytofluorimetric analysis of CD44^high^/CD24^low^ stem/progenitor population in ctrl/shBeta MCF7 cells upon Nor/1%pO_2_ conditions; **D**, Real-Time PCR analysis of ESR1 mRNA level in Ctrl/shBeta MCF7 cells upon Nor/1%pO_2_ conditions; **E**, Immunofluorescence (IF) analysis of Beta-catenin in Nor/1%pO_2_ MCF7 cells; **F**, WB analysis of beta-catenin in Nor/1%pO_2_ MCF7 cells cytoplasmic and nuclear fractions (lamin B and beta-tubulin were used as fractionation controls); **G**, beta-catenin/TCF transcriptional reporter (TOPFLASH) assay in MCF7 cells and T-MS under Nor/1%pO_2_. Data are presented as mean +/- s.d.; p values refers to t test. n=3, unless otherwise specified.

We then investigated the role of beta-catenin in the regulation of the CA9 and SNAI2-dependent breast cancer stem cell phenotype. MCF7 cells carrying beta-catenin specific shRNA retroviral vector (shBeta) displayed a dramatic reduction of SNAI2 and CA9 protein expression (Figure 2A), coupled with reduced normoxic MS formation and impaired hypoxic MS expansion (Figure 2B). Breast cancer stem/progenitor cells are also over-represented in the CD44^high^/CD24^low^ sub-population [40]. Consistent with the data on MS, MCF7-shBeta cells disclosed curtailed proportion of CD44^high^/CD24^low^ cells in normoxia, and blunted CD44^high^/CD24^low^ population expansion under hypoxia (Figure 2C). In long-term hypoxia-exposed MCF7-shBeta cells, we also observed decreased ability to form foci (Figure S2A). Moreover shRNA mediated beta-catenin knockdown remarkably reduced soft agar colony formation capability (Figure S3A), this latter being a stringent *in vitro* assay for detecting cell malignant transformation. These data led us to reason that beta-catenin knockdown hampers stem/progenitor cell self-renewal. Interestingly, beta-catenin knockdown also hindered the hypoxia-induced down-regulation of ESR1 (Figure 2D), revealing the capability of beta-catenin to play a pivotal role in the hypoxia-induced de-differentiation program that parallels the gain of stem cell features in breast cancer cells. We then observed that hypoxia elicited substantial delocalization of beta-catenin from the cell membrane to the cytoplasm, this fact being paralleled by a reduction in cell-to-cell contacts (Figure 2E). Interestingly, hypoxia triggered neither beta-catenin nuclear localization nor beta-catenin/TCF transcriptional activity in MCF7 cells and in T-MS (Figure 2F, G). These data led us to conceive that beta-catenin facilitates CA9 and SNAI2 expression and the ensuing stem cell phenotype in hypoxia-exposed breast cancer cells, independently of its nuclear transcriptional activity.

### Hypoxia induces CA9 and SNAI2 expression via HIF1-alpha dependent mRNA transcription and beta-catenin dependent mRNA stabilization

**Figure 3:**
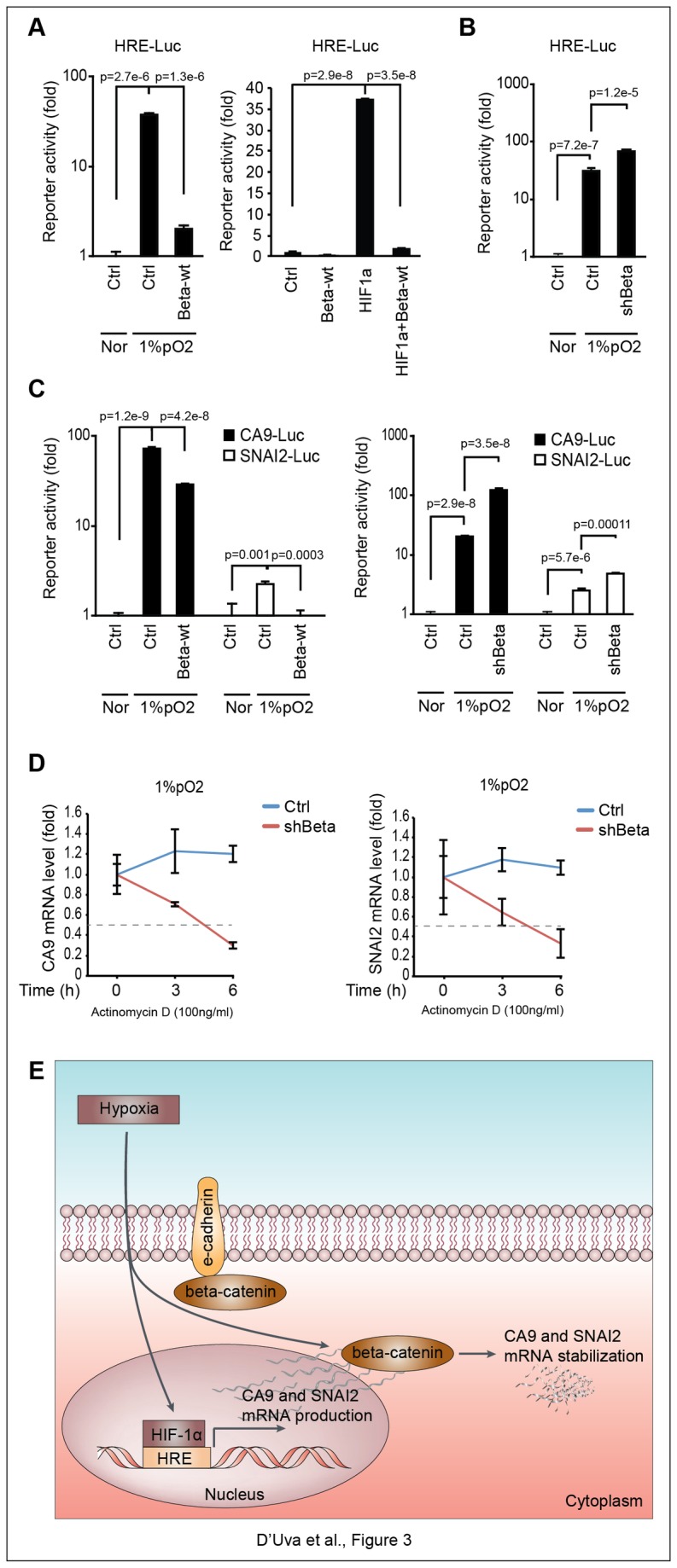
Hypoxia induces CA9 and SNAI2 expression via HIF1-alpha dependent mRNA production and beta-catenin dependent stabilization. **A**, HIF-1alpha transcriptional reporter (HRE-Luc) assay in MCF7 cells transfected with wild type beta-catenin (Beta-wt) under Nor/1%pO_2_ conditions or in combination with HIF1-alpha (HIF1a) encoding vector; **B**, HRE-Luc assay in 1%pO_2_-exposed ctrl/shBeta MCF7 cells; **C**, CA9-Luc and SNAI2-Luc assay in ctrl/Beta-wt transfected and in ctrl/shBeta MCF7 cells under Nor/1%pO_2_ conditions; **D**, CA9 and SNAI2 mRNA stability assay following inhibition of Polymerase 2 transcriptional activity by actinomycin D (100ng/ml) in ctrl/shBeta MCF7 cells exposed to 1%pO_2_; **E**, Schematic representation of the HIF1-alpha/beta-catenin interplay in breast cancer cells in response to hypoxia: HIF1-alpha promotes transcription and cytoplasmic beta-catenin enhances stabilization of SNAI2 and CA9 mRNAs; the negative effect of beta-catenin on HIF-1alpha-induced transcription is also depicted. Data are presented as mean +/- s.d.; p values refers to t test. n=3, unless otherwise specified.

CA9 and SNAI2 are hypoxia-inducible-factor-1-alpha (HIF-1alpha) transcriptional targets [8,20]. Recently, it has been suggested that beta-catenin promotes CA9 expression, by acting as HIF-1alpha transcriptional co-factor in colon cancer cells [8]. Prompted by these data, we sought to investigate the effect of beta-catenin on HIF-1alpha-dependent transcription, as well as on CA9 and SNAI2 promoter activity. Surprisingly, the luciferase driven responsive reporter assay demonstrated that beta-catenin over-expression hampers HIF1-alpha transcriptional activity upon hypoxia exposure or following HIF1-alpha transient overexpression (Figure 3A). Consistently, beta-catenin knockdown triggered HIF1-alpha transcriptional activity (Figure 3B). Similarly, beta-catenin suppressed the hypoxia-induced CA9 and SNAI2 genes promoter activity (Figure 3C). These findings point to the onset of antagonistic activity between beta-catenin and HIF1-alpha mediated transcription in hypoxia-exposed breast cancer cells. In line with these results, the transfection of the dominant negative isoform of TCF4 (TCF4-DN), which halts beta-catenin transcriptional activity [6], was not able to decrease CA9 and SNAI2 mRNA expression in hypoxia-exposed MCF7 cells (Figure S3B). Evidence that beta-catenin paradoxically reduces CA9 and SNAI2 promoter activity, but increases the expression levels of cognate mRNA, prompted us to investigate mRNA stability as a new layer of beta-catenin-dependent function. To prove this hypothesis, we used actinomycin D, an inhibitor of Polymerase 2 (Pol2) that impairs *de novo* mRNA transcription. In line with our hypothesis, stable beta-catenin silencing shortened CA9 and SNAI2 mRNA half-life in hypoxia exposed MCF7 cells (Figure 3D). These data suggest that the hypoxia-induced de-differentiation/stem cell program in breast cancer cells relies upon the HIF1alpha-dependent production of CA9 and SNAI2 mRNA, followed by the beta-catenin dependent stabilization of these mRNAs (Figure 3E). 

### Constitutively active beta-catenin post-transcriptional activity in basal-like/triple-negative breast cancer cells

**Figure 4:**
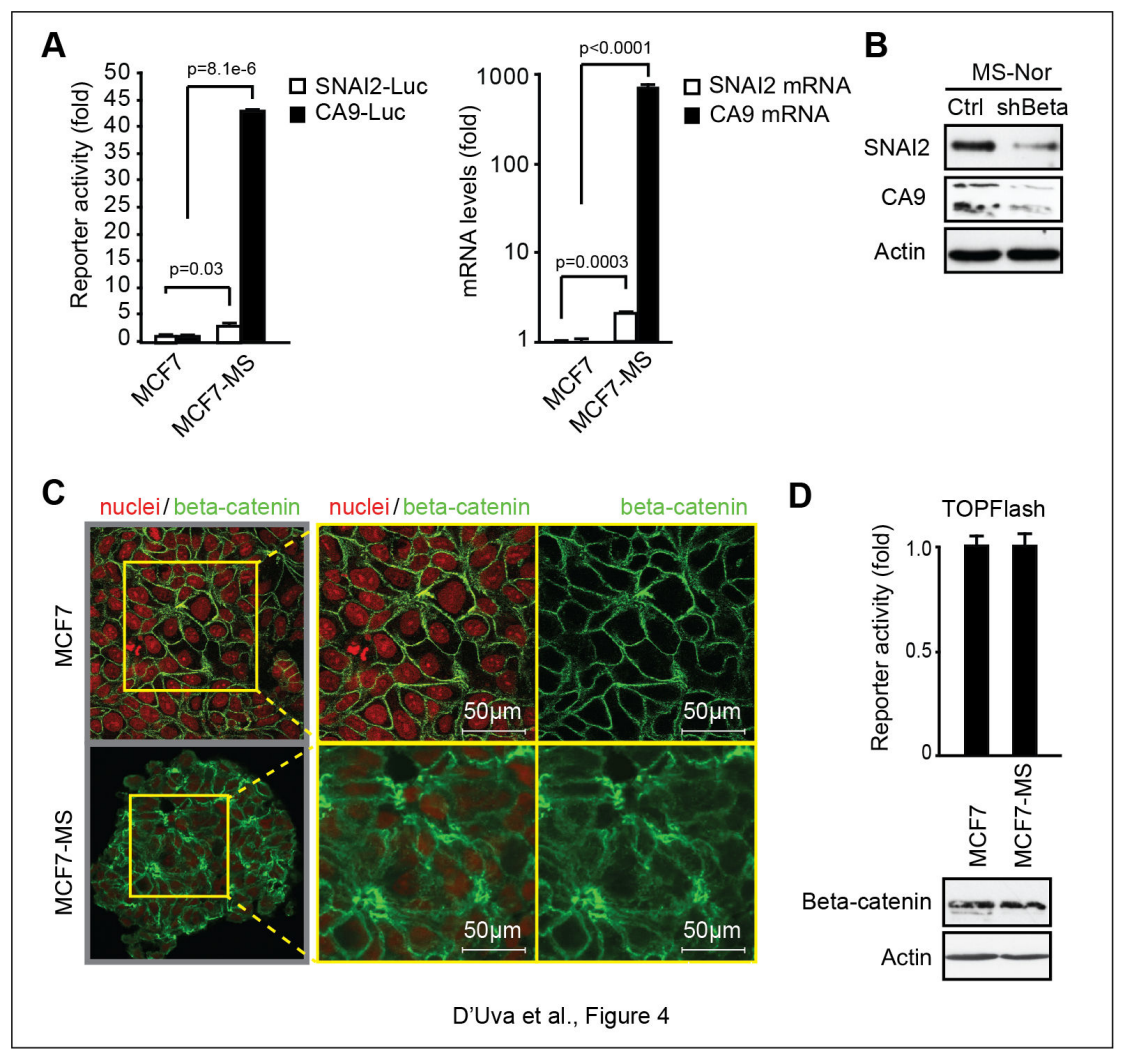
Beta-catenin maintains the stem/progenitor cell pool in normoxia, independently of its nuclear transcriptional activity. **A**, CA9 and SNAI2 Real Time PCR analysis and CA9-luc and SNAI2-Luc promoter activity in adherent MCF7 cells and MCF7-MS; **B**, WB analysis of SNAI2 and CA9 in MCF7-MS; **C**, beta-catenin IF analysis in adherent MCF7 cells and MCF7-MS; **D**, TOPFLASH assay and WB analysis of beta-catenin in adherent MCF7 cells and MCF7-MS. Data are presented as mean +/- s.d.; p values refers to t test. n=3, unless otherwise specified.

**Figure 5:**
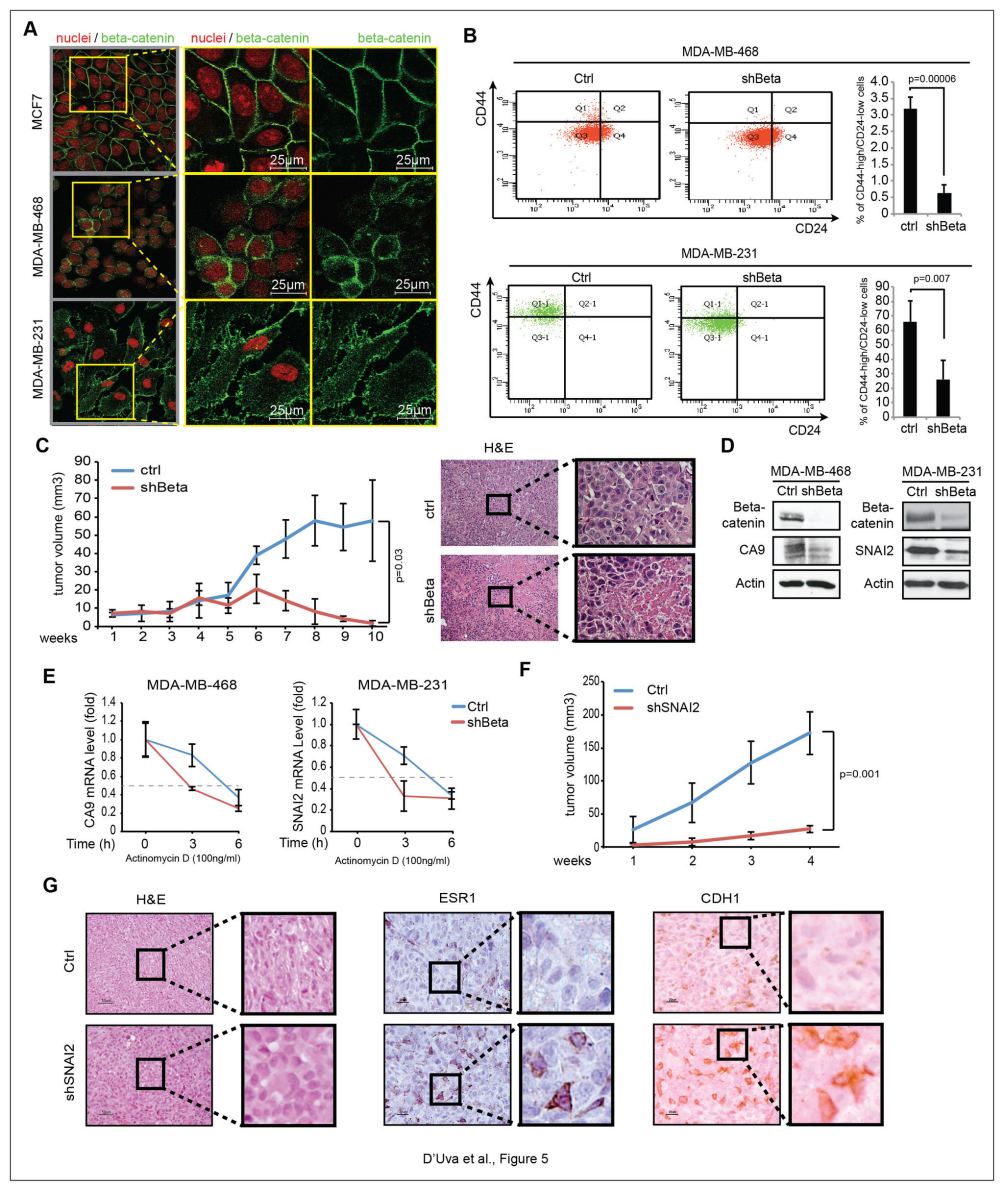
In normoxic basal-like breast cancer cells, cytoplasmic beta-catenin promotes stem cell features *in vitro* and tumor growth *in vivo* via constitutive stabilization of CA9 and SNAI2 mRNAs. **A**, Beta-catenin IF analysis in luminal MCF7 cells and in basal-like MDA-MB-468 and MDA-MB-231 cells; **B**, Cytofluorimetric analysis of the CD44^high^/CD24^low^ population in ctrl/shBeta MDA-MB-468 and MDA-MB-231 cells; **C**, Ctrl/shBeta MDA-MB-468 10-weeks mammary fat pad xenograft assay (n=5, each group); representative pictures of xenograft tissues hematoxylin-eosin staining are include; **D**, WB analysis of CA9, SNAI2 and beta-catenin protein levels in ctrl/shBeta normoxic MDA-MB-468 and MDA-MB-231 cells; **E**, CA9 and SNAI2 mRNA stability assay following inhibition of Polymerase 2 transcriptional activity by actinomycin D (100ng/ml) in Ctrl/shBeta MDA-MB-468 and MDA-MB-231 cells; **F**, Four weeks growth curve of ctrl/shSNAI2 MDA-MB-231 subcutaneous xenograft assay (n=6, each group); **G**, Hematoxylin-eosin, ESR1 and CDH1 immunohistochemical stainings in xenograft tissue sections. Data are presented as mean +/- s.d.; p values refers to t test. n=3, unless otherwise specified.

 When we compared MCF7 derived MS to cognate adherent cells for CA9 and SNAI2 expression, we found higher CA9 and SNAI2 promoter activity and mRNA expression that was halted in shBeta normoxic MS (Figure 4A-B). Albeit the phenomenon was paralleled by the increase in beta-catenin cytoplasmic localization (Figure 4C), similar levels of total beta-catenin protein and transcriptional activity were present in adherent and MCF7 derived MS (Figure 4D). These data point out that the post-transcriptional activity of beta-catenin might be involved in the maintenance of the stem/progenitor cell status, even in normoxia. Following this reasoning, we drew our attention to literature supporting the notion that cancer stem cell features are overtly represented in basal-like breast cancer cells and tissues [27,28]. Interestingly, in this aggressive tumor subtype, cytoplasmic beta-catenin localization has recently been observed [29–31]. Therefore, we sought to analyze the functional relationship between beta-catenin post-transcriptional activity and the cancer stem cell phenotype in MDA-MB-468 and MDA-MB-231 basal-like breast cancer cell lines [35]. In these cells we first observed the constitutive cytoplasmic beta-catenin localization in normoxia (Figure 5A). Then, in keeping with expectations, we found that the shRNA mediated beta-catenin knockdown remarkably reduced the extent of CD44^high^/CD24^low^ stem/progenitor cell population (Figure 5B). Moreover, we observed that MDA-MB-468-shBeta xenografts were characterized by reduced growth rate and by the presence of eosinofilic necrotic areas enriched with cells showing hyper-chromatic and pleomorphic picnotic nuclei (Figure 5C). In line with these features, beta-catenin knockdown reduced CA9 and SNAI2 protein expression (Figure 5D) and mRNA stability (Figure 5E). With regard to this issue, overtly blunted tumor growth rate was found in MDA-MB-231 SNAI2-shRNA cells (Figure 5F). SNAI2 knockdown xenografts disclosed the increase in epithelia-like cell morphology and the re-expression of the breast tissue differentiation markers ER-alpha and E-cadherin (Figure 5G). Since that CA9 knockdown halts MDA-MB-231 xenografts [22], the available data agree on the crucial role of the beta-catenin-dependent CA9 and SNAI2 mRNA stability and expression in the biology of basal-like/triple-negative breast cancer.

### Beta-catenin binds and stabilizes CA9 and SNAI2 mRNAs 3’-UTR and facilitates the shift of HuR/mRNA complexes to the 40S ribosome subunit

We then aimed at analyzing the molecular mechanism through which beta-catenin exerts its post-transcriptional control on CA9 and SNAI2 mRNAs. Cytoplasmic beta-catenin has recently been reported to affect the stability of cyclooxygenase-2 (COX2) mRNA by binding the mRNA 3’ un-translated regions (3’-UTRs), in co-operation with the mRNA binding protein HuR [14,15,17]. 

**Figure 6:**
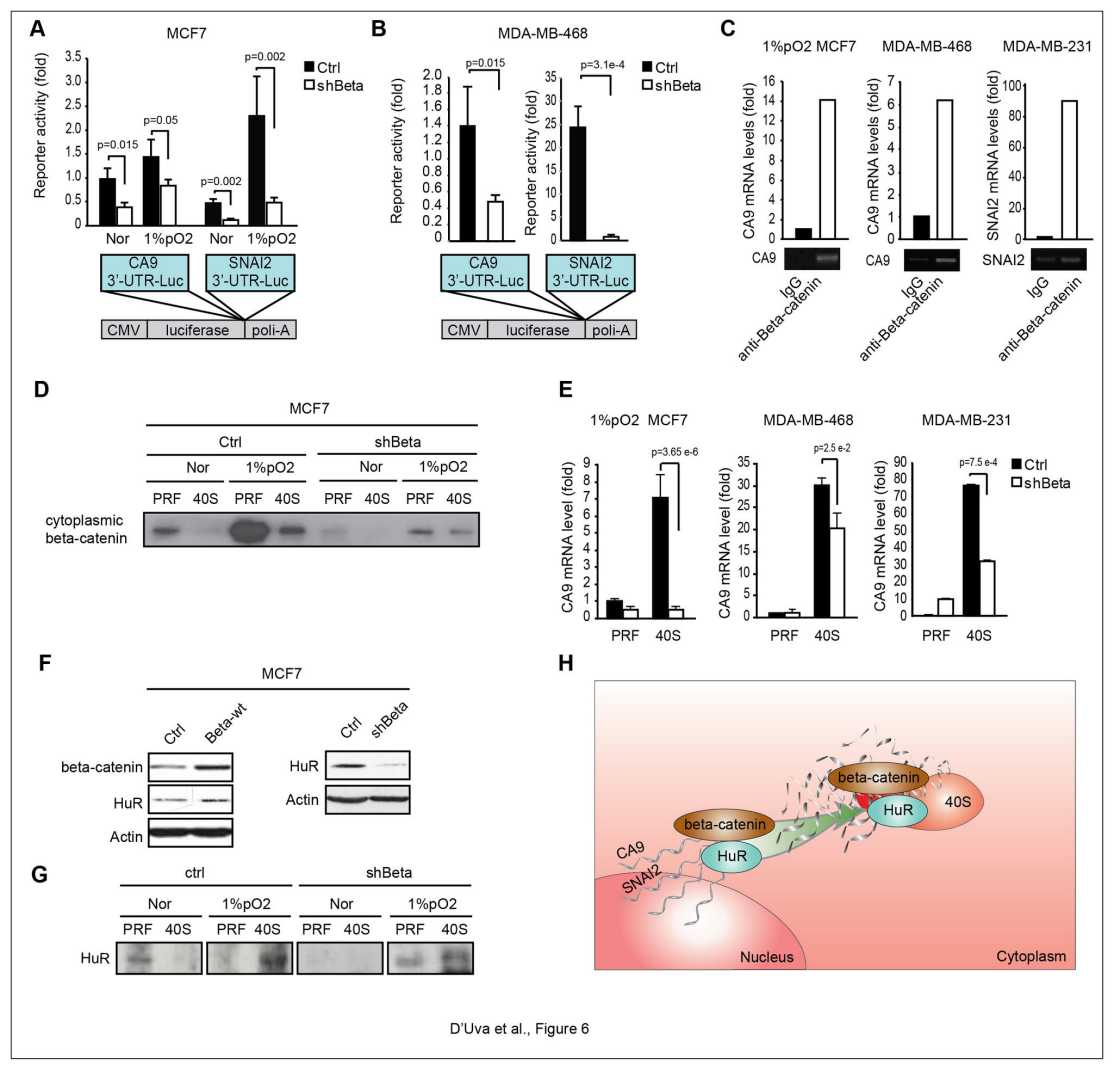
Beta-catenin stabilizes CA9 and SNAI2 mRNAs through direct binding and facilitating the shift of HuR/mRNA complexes to the ribosomal compartment. **A**, CA9 and SNAI2 3’UTR-luciferase reporter (CA9-3’UTR-Luc and SNAI2-3’UTR-Luc) assay in ctrl/shBeta MCF7 cells exposed to Nor/1%pO_2_ conditions; **B**, CA9 and SNAI2 3’UTR-luciferase reporter assay in ctrl/shBeta MDA-MB-468 cells; Schematic diagram of the CA9 or SNAI2 3’UTR luciferase encoding vectors carrying either CA9 or SNAI2 3’UTR sequences inserted between the luciferase coding sequence and the polyadenylation site; **C**, Quantitative CA9 and SNAI2 mRNA immunoprecipitation assay with mouse IgG/beta-catenin antibody in 1%pO_2_ MCF7 cells, MDA-MB-468 and MDA-MB-231 cells; **D**, WB analysis of cytoplasm pre-ribosomal (PRF) and 40S ribosomal (40S) cytoplasmic fractions of ctrl/shBeta MCF7 upon exposure to Nor/1%pO_2_ conditions; total beta-catenin protein levels are reported in Figure 2A and S5A; E, Real-Time PCR analysis of CA9 mRNA levels in PRF/40S cytoplasmic fractions of 1%pO_2_ exposed ctrl/shBeta MCF7, MDA-MB-468 and MDA-MB-231 cells; **F**, WB of beta-catenin and HuR protein levels in ctrl/beta-wt and in ctrl/shBeta MCF7 cells; **G**, WB analysis of HuR in PRF and 40S cytoplasmic fractions of ctrl/shBeta MCF7 cells exposed to Nor/1%pO_2_; note that total HuR protein levels of Nor/1%pO_2_ MCF7 cells refer to Figure S5D; H, Schematic representation of the cytoplasmic beta-catenin/HuR post-transcriptional machinery in the regulation of CA9 and SNAI2 mRNAs, via stabilization, direct binding and shuttling to the ribosomal compartment. Data are presented as mean +/- s.d.; p values refers to t test. n=3, unless otherwise specified.

To evaluate whether the beta-catenin dependent increase in CA9 and SNAI2 mRNA stability relies on their 3’-UTRs, we examined luciferase encoding vectors carrying either CA9 or SNAI2 3’UTR sequences inserted between the luciferase coding sequence and the poly-adenylation site. Following this approach, we observed hypoxia-induced increase in the CA9 and SNAI2 3’UTR luciferase reporters activity, which was significantly reduced in normoxic and hypoxic MCF7-shBeta cells, compared to controls (Figure 6A). These data indicate that beta-catenin promotes CA9 and SNAI2 mRNA stability via their 3’UTR sequences and that this phenomenon was triggered by hypoxia. These findings were then confirmed in normoxic basal-like cells, in which we observed the reduction of CA9 and SNAI2 luciferase 3’UTR reporter activity in shBeta cells (Figure 6B, S3C). Then, the mRNA immuno-precipitation assay revealed that SNAI2 and CA9 mRNA were present in beta-catenin immune-precipitates from hypoxic MCF7 cells, normoxic MDA-MB-468 and MDA-MB-231 cells, and hypoxic T-MS (Figure 6C, S3D). These data provide compelling evidence for the direct binding of beta-catenin to SNAI2 and CA9 mRNAs. Interestingly, bioinformatics analysis showed that SNAI2 and CA9 mRNA 3-UTR’s contain U/AU-rich sequences (Figure S4A), which represent acknowledged HuR binding sites [41–44]. In keeping with the expectations, SNAI2 and CA9 mRNA were amplified from HuR immune-precipitates (Figure S4B). Moreover, the siRNA-mediated HuR knockdown blunted the expression of CA9 and SNAI2 mRNAs (Figure S4C). These data suggest that HuR is part of the beta-catenin post-transcriptional machinery, which promotes CA9 and SNAI2 mRNAs stabilization. 

HuR ensures timely engagement of mRNA with ribosomes, a function which remains poorly characterized [42]. The analysis of density gradient-separated cytosolic fractions of MCF7 cells conveyed that the exposure to hypoxia elicited the delocalization of beta-catenin to the cytoplasmic and 40S ribosome compartments, and that the phenomenon was substantially reduced in shBeta cells (Figure 6D). Intriguingly, constitutive beta-catenin localization in the ribosomal compartment was observed in normoxic basal-like cells (Figure S5A-S5B). Because beta-catenin binds cytoplasmic mRNA and localizes to the ribosomal compartment, we assessed whether beta-catenin plays a role in the transport of CA9 and SNAI2 mRNA to these cytoplasmic structures. We found that beta-catenin knockdown substantially reduced the localization of CA9 mRNA to the 40S ribosomal compartment (Figure 6E), albeit the phenomenon was not significant for SNAI2 mRNA (Figure S5C). We then observed that beta-catenin up-regulated HuR expression (Figure 6F), suggesting that beta-catenin increases the stabilization of target mRNAs, by increasing HuR levels. Moreover, beta-catenin knockdown hindered the hypoxia-induced translocation of HuR to the 40S ribosome compartment (Figure 6G). In line with these data, beta-catenin shRNA reduced the constitutive localization of HuR to the 40S ribosome compartment in basal-like cells (Figure S5D). Overall, our data suggest that cytoplasmic beta-catenin post-transcriptionally regulates CA9 and SNAI2 mRNAs in hypoxic luminal and normoxic basal-like cells, either by direct binding cytoplasmic mRNAs or by facilitating the shuttling of HuR/mRNA complexes to the 40S ribosome sub-unit (Figure 6H).

### Beta-catenin post-transcriptionally regulates a subset of EGF-regulated mRNAs

**Figure 7:**
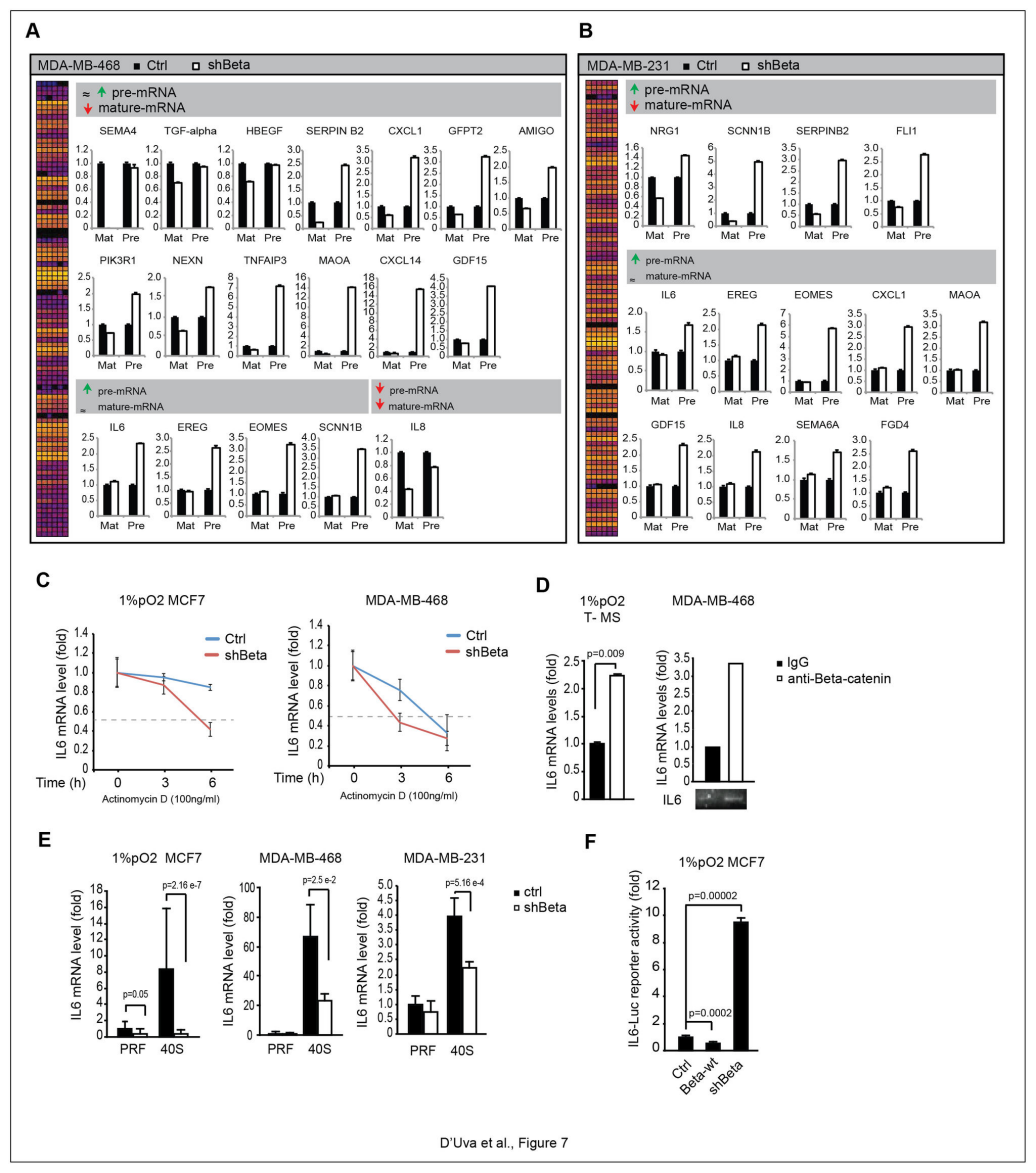
Involvement of the beta-catenin-driven machinery in the stabilization of EGF-induced mRNAs. **A**, **B**, Fluidigm® Real-Time PCR analysis of EGFR pathway in ctrl/shBeta MDA-MB-468 and MDA-MB-231 cells. The assay distinguishes mature-mRNA and immature (pre-mRNA) levels; **C**, IL6 mRNA stability assay following Polymerase 2 transcriptional activity inhibition by actinomycin D (100ng/ml) in 1%pO_2_ exposed ctrl/shBeta MCF7 and MDA-MB-468 cells; **D**, Quantitative IL6 mRNA immunoprecipitation assay with mouse IgG/beta-catenin antibody in 1%pO_2_ T-MS (Real-Time PCR) and normoxic MDA-MB-468 cells (Standard RT-PCR); **E**, Real-Time PCR of IL6 mRNA levels in PRF and 40S cytoplasmic fractions in Nor/1%pO_2_ MCF7, MDA-MB-468 and MDA-MB-231 cells; **F**, IL6 promoter-driven luciferase (IL6-Luc) assay in Ctrl, Beta-wt and 1%pO_2_ exposed shBeta MCF7 cells. Data are presented as mean +/- s.d.; p values refers to t test. n=3, unless otherwise specified.

A peculiar feature of basal-like/triple-negative tumors is the over-expression of epidermal growth factor receptor (EGFR) [45,46], a receptor which plays a pivotal role in cancer development [47,48]. EGFR is capable of modulating beta-catenin intracellular localization [49,50]. Both SNAI2 and CA9 are EGFR downstream targets [51–53]. We observed that the stable over-expression of wild-type EGFR in MCF7 cells raised the level of cytoplasmic beta-catenin levels, a phenomenon further augmented by EGF administration (Figure S6A). Moreover, wild-type EGFR overexpression triggered the beta-catenin dependent up-regulation of CA9 and SNAI2 mRNA expression (Figure S6B). Following these data, we extended our observations to other genes, which might be involved in the beta-catenin dependent post-transcriptional mechanism described above. We went on to analyze the expression level of 34 EGF-induced mRNAs via high throughput Fluidigm System (Table S1). In order to discriminate between transcriptional and post-transcriptional targets of beta-catenin, each gene was measured for its precursor mRNA (pre-mRNA) and its mature mRNA expression levels, via amplification of intronic and exonic sequences [54]. In line with our predictions, no significant down-regulation of pre-mRNA level occurred in shBeta basal-like cells (Figure 7A-B), with the unique exception of IL8, which had been already described as a beta-catenin transcriptional target [55]. We identified genes that underwent beta-catenin dependent stabilization: seventeen in MDA-MB-468 and thirteen MDA-MB-231 cells. Two subsets were identified: the first contained genes displaying reduced mature mRNA levels, coupled with unchanged or increased levels of pre-mRNA; the second contained genes characterized by unchanged mature mRNA levels and increased pre-mRNA levels. Incidentally, the increase of certain pre-mRNA led to hypothesize the existence of transcriptional compensatory mechanisms, consequent to reduced mRNA stability, reminiscent of the increase in CA9 and SNAI2 promoter reporter activity observed in shBeta cells (see Figure 3B). Striking overlapping of beta-catenin regulated genes was observed between the two cell lines. In order to validate the Fluidigm data, we focused our attention on the pivotal breast cancer stem cells growth factor Interleukin 6 (IL6) [21,34]. We were able to ascertain that beta-catenin knockdown reduced IL6 mRNA stability in hypoxic MCF7 cells and normoxic MDA-MB-468 cells (Figure 7C). As with CA9 and SNAI2, we found that beta-catenin was bound to IL6 mRNA in hypoxic luminal and normoxic basal-like cells (Figure 7D), and facilitated the shuttling of IL6 mRNA to the 40S ribosomal compartment (Figure 7E). Furthermore, the expected compensatory increase of IL6 promoter activity was observed in shBeta cells (Figure 7F). These data demonstrate that the increase in IL6 mRNA level occurs independently of the beta-catenin transcriptional activity. Finally, bio-informatics analysis revealed that IL6 mRNA 3-UTR’s sequence harbored U/AU-Rich sequences (Figure S7A), and that HuR knockdown almost extinguished IL6 gene expression (Figure S7B). Collectively, our data suggest that beta-catenin post-transcriptionally promotes mRNA stability of a large subset of EGFR-regulated genes, which share in stem cell regulation and part of the basal-like/triple-negative phenotype. 

## Discussion and Conclusions

Comprehension of the post-transcriptional mechanisms that steers stem cell features and cancer malignancy represents cutting edge cancer research. Recent studies described a new role of beta-catenin in the post-transcriptional regulation of several cytoplasmic mRNAs [13–17], in addition to its well-established role in promoting specific gene expression [6]. Here we provide evidence that this still-poorly characterized beta-catenin activity impacts the breast cancer stem cell phenotype upon exposure to hypoxia. In particular, our study demonstrates that, in response to hypoxia, the beta-catenin/HuR post-transcriptional machinery reduces the differentiation and boosts cancer stem cell features via increased mRNA stabilization of the stem cell regulator SNAI2 and the hypoxia survival CA9 gene.

At the transcriptional level, our data support the notion that hypoxia and its mediator HIF-1alpha do not cooperate with beta-catenin transcriptional activity. These data are in line with previous observations in colon and lung cancer cells [8,56], but nevertheless in contrast to observations in embryonic stem cells [9]. In particular, beta-catenin even represses HIF-1alpha transcriptional activity, and the hypoxic transcriptional up-regulation of the HIF-1alpha targets CA9 and SNAI2. Based on the reasoning above, our data suggest that the expression of breast cancer stem cell regulatory genes requires HIF-1-alpha dependent mRNA production, followed by beta-catenin dependent stabilization of the same mRNAs. The reciprocal transcriptional inhibition between beta-catenin and HIF1-alpha may take part in the negative feedback loop, which finely tunes transcriptional and post-transcriptional mechanisms. 

 Cytoplasmic beta-catenin stabilizes Cox-2 mRNA through physical interaction with its 3’-UTRs [14,15,17]. In line with these findings, we demonstrated that beta-catenin binds and stabilizes CA9, SNAI2 and IL6 mRNAs via their 3’-UTR sequences. We also show that, at least for CA9 and IL6 mRNA, beta-catenin facilitates the shift to the 40S ribosomal compartment. The interaction between beta-catenin and cytoplasmic mRNA involves also HuR [14,15,17], a protein that binds and stabilizes U/AU-Rich mRNAs [41–44]. HuR expression correlates with poor outcome in breast cancers [57]. Here we show that HuR directly binds with and promotes the expression of CA9, SNAI2 and IL6 mRNAs. Physical interaction between HuR and beta-catenin has been previously reported [14,15,17]. In agreement with these reports, we detected a minor fraction of beta-catenin in HuR immune-precipitates (Figure S8A). In regard to this issue, we also report that stable beta-catenin silencing reduces HuR protein levels and its localization to the 40S ribosomal subunit, suggesting that beta-catenin facilitates the shift of HuR/mRNA complexes to the 40S ribosomal compartment. Enhanced mRNA stability, coupled with reduced transcriptional activity, is likely to be an appropriate strategy for maintaining specific gene expression profiles under energy restrictive conditions, such as hypoxia. 

 Basal-like/triple-negative tumors are aggressive breast cancers characterized by the expression of a stem cell gene profile [27,28]. Interestingly, cytoplasmic beta-catenin localization was specifically observed in basal-like tissues [29,31]. Over-expression of CA9 and SNAI2 genes has been associated with this breast cancer sub-type [19,32,33]. Here we show that in basal-like/triple-negative breast cancer cells, beta-catenin/HuR post-transcriptional machinery operates even under normoxic conditions, promoting CA9 and SNAI2 gene mRNA stability and expression. The knockdown of CA9 and SNAI2 substantially slows down *in vivo* tumor growth of basal-like/triple-negative breast cancer cells [22] (this investigation). Speculatively, in basal-like cells, the trigger effect of hypoxia on beta-catenin post-transcriptional machinery may be mimicked by specific genetic alterations, e.g. EGFR over-expression [45,46]. EGFR plays a pivotal role in cancer [47,48] and EGFR-targeted agents are among the therapeutic agents being actively investigated in clinical trials in patients with triple-negative/basal-like breast cancers [46]. Activation of the EGFR pathway modifies beta-catenin intracellular localization [49,50], and controls the homeostasis of normal and malignant mammary gland stem cells [58]. In that regard we observed elevated cytoplasmic beta-catenin level in EGFR over-expressing MCF7 cells (Figure S6A). Interestingly, we also detected increased EGFR levels and increased beta-catenin cytoplasmic localization in cells transfected with p53-inactivating mini-protein (Figure S6C). Since p53 mutations are very common in triple negative/basal-like breast cancer [59], this observation suggests that p53 potentially may contribute to the onset of the basal-like/stem phenotype in breast cancer cells triggering beta-catenin post-transcriptional activity. We also provide evidence that beta-catenin post-transcriptionally enhances the expression of an array of EGFR-controlled genes. This observation is in line with the previously reported enrichment in AU-Rich unstable mRNAs among the EGFR signaling pathway target mRNAs [51]. Interestingly, we observed a beta-catenin dependent regulation of several pro-inflammatory cytokines (IL6, IL8, CXCL1, CXCL14). In particular, we here characterized the beta-catenin post-transcriptionally regulation of IL6, In addition, IL8 mRNA immuno-precipitation and stabilization by beta catenin in breast cancer stem cells was recently reported by our group [60]. Both IL6 and IL8 are crucial regulators of breast cancer stem cell growth and survival [21,34,61] and are part of the cancer stem cells pro-inflammatory phenotype [62,63]. Owing to the role of HuR in the initiation and resolution of inflammation [64], our study suggests that the beta-catenin/HuR post-transcriptional machinery regulates the inflammatory phenotype of EGFR over-expressing cancers, such as triple negative/basal-like tumors [46]. 

The expression of CD44, the hallmark of breast cancer stem cells, is transcriptionally promoted by hypoxia, via HIF-1alpha activation [20,65]. Here we observed that the expression of CD44 is also post-transcriptionally regulated by beta-catenin, in similarity to CA9, SNAI2 and IL6. In particular, shBeta cells show reduced CD44 mRNA stability (Figure S9A) and impaired CD44 mRNA translocation to the 40S ribosome compartment (Figure S9B). Interestingly, HuR, but not beta-catenin, binds CD44 mRNA (Figure S9C), and HuR knockdown blunts the expression of CD44 gene (Figure S9D). These data suggest that beta-catenin dependent post-transcriptional regulation occurs to a large set of genes, which harbor U/AU-Rich motifs at their 3’UTR, and which cooperate in the set up of the stem cell phenotype. 

In conclusion, our study highlights the role of post-transcriptional mechanisms in the regulation of cancer stem cell features and cancer aggressiveness, and leads to reason that interfering with the beta-catenin post-transcriptional activity may be an innovative strategy to target breast cancer stem cells and to treat aggressive basal-like breast cancer patients.

## Supporting Information

Figure S1 **Hypoxia reduces proliferation and differentiation in MCF7 cells, and elicits Snai2 dependent stem cell features in MCF7 cells and T-MS.** **A**, SNAI2 and CA9 promoter luciferase reporter (SNAI2-Luc and CA9-Luc) assay in Nor/1%pO2 MCF7 cells and T-MS; B, T-MS formation upon ctrl/SNAI2-specific siRNA(siSNAI2) transfection of breast cancer primary cells exposed to 1%pO_2_; **C**, WB analysis of e-cadherin protein level in SNAI2-specific shRNA retroviral vector (shSNAI2) MCF7 cells upon Nor/1%pO2 conditions.
(TIF)

Figure S2 **beta-catenin knockdown reduces focus forming capability in hypoxia-exposed MCF7 cells.** **A**, Focus assay in 1%pO_2_ long-term-exposed (2 weeks) ctrl/shBeta MCF7 cells.
(TIF)

Figure S3 **beta-catenin role in breast cancer cell proliferation, colony-forming ability in soft agar and in CA9 and Snai2 transcriptional and post-transcriptional regulation.** A, soft agar assay in ctrl/shBeta MDA-MB-231 cells; **B**, RT-PCR analysis of CA9 and SNAI2 mRNA level in 1%pO_2_-exposed MCF7 cells transfected with ctrl/TCF4DN; **C**, SNAI2-3’UTR-Luc assay in ctrl/shBeta MDA-MB-231 cells; **D**, quantitative CA9 and SNAI2 mRNA immunoprecipitation assay by control IgG/anti-beta-catenin antibody.
(TIF)

Figure S4 **HuR binds and stabilizes CA9 and SNAI2 mRNAs in hypoxic luminal and normoxic basal-like breast cancer cells.** **A**, schematic representation of CA9 and SNAI2 mRNA 3’-UTRs HuR binding sites as predicted by bio-informatics analysis; **B**, quantitative CA9 and SNAI2 mRNA immunoprecipitation assay by control IgG/beta-catenin antibody; **C**, Real Time PCR analysis of SNAI2 and CA9 mRNA levels in Ctrl siHuR-transfected/1%pO2-exposed MCF7 cells and normoxic MDA-MB-468 cells.
(TIF)

Figure S5 **beta-catenin knock-down reduces HuR expression and localization to the ribosomal compartment.** **A**, WB analysis of beta-catenin protein levels in MCF7 cells exposed to 1%pO_2_; **B**, WB analysis of beta- catenin protein levels in PRF and 40S cytoplasmic fractions of ctrl/shBeta MDA-MB-468 and MDA-MB-231 cells; **C**, Real Time PCR analysis of SNAI2 mRNA levels in PRF/40S cytoplasmic fractions of ctrl/shBeta 1%pO_2_ MCF7 and MDA-MB-231 cells; **D**, WB analysis of HuR protein levels in total cell lysates and in PRF/40S cytoplasmic fractions of ctrl/shBeta MDA-MB-468 and MDA-MB-231 cells; note that actin protein levels of ctrl/shBeta MDA-MB-468 cells refers to Figure 5D.
(TIF)

Figure S6 **EGFr overexpression and activation promotes the cytoplasmic localization of beta-catenin and the beta-catenin dependent increase in SNAI2 and CA9 mRNA expression.** **A**, IF analysis of beta-catenin in MCF7 cells stably-transfected with empty (MCF7-ctrl) or wild-type EGFR (MCF7-EGFr) vector, in presence/absence of EGF (10ng/ml; 24h); **B**, RT-PCR analysis of CA9 and SNAI2 mRNA expression levels in MCF7-ctrl/MCF7-EGFR and in MCF7-EGFR cells, transiently transduced with ctrl/shBeta encoding vectors; **C**, IF analysis of beta-catenin in p53-dominant-negative (p53D) stably transfected MCF7 cells; note that IF of MCF7-ctrl cells refers to panel A; **D**, WB analysis of EGFR protein levels in MCF7-ctrl/MCF7-p53D.
(TIF)

Figure S7 **HuR binds and stabilizes IL6 mRNA.** **A**, schematic representation of IL6 3’-UTR HuR binding sites as predicted by bioinformatics analysis; **B**, RT-PCR analysis of IL6 mRNA levels in 1%pO_2_ MCF7 cells, transiently transfected with Ctrl/siHuR; note that the loading control (28S ribosomal subunit mRNA) of ctrl/siHuR 1%pO_2_ MCF7 cells refers to Figure S4C.
(TIF)

Figure S8 **Beta-catenin/HuR physically interacts.** **A**, Co-immunoprecipitation assay of beta-catenin and HuR proteins in MCF7, MCF7-MS and MDA-MB-231 cells.
(TIF)

Figure S9 **Beta-catenin/HuR post-transcriptional machinery stabilizes CD44 mRNA.** **A**, CD44 mRNA stability assay (actinomycin D, 100ng/ml) in ctrl/shBeta MDA-MB-468 and MDA-MB-231 cells; **B**, Real Time PCR analysis of CD44 mRNA levels in PRF/40S cytoplasmic fractions of ctrl/shBeta 1%pO_2_ MCF7, MDA-MB-468 and MDA-MB-231 cells; **C**, quantitative CD44 mRNA immunoprecipitation assay with control IgG/anti-HuR/anti-beta-catenin antibody in 1%pO_2_ MCF7 cells and MDA-MB-468 cells; **D**, RT-PCR analysis of CD44 mRNA levels in scr/siHuR transfected MCF7 cells, exposed to 1%pO_2_; note that the loading control (28S ribosomal subunit mRNA) of ctrl/shBeta 1%pO_2_ MCF7 cells refers to Figure S4C.
(TIF)

Material S1 **Cytoplasmic pre-ribosomal and ribosome fractionation.** **A**, profile of cytoplasmic fractions obtained after centrifugation of cytoplasmic lysates; **B**, fractions corresponding to low density pre-ribosomal cytoplasm (PRF), 40S, 60-80S and polysomes were pooled and examined in 1% agarose gel and western blot to verify the presence of the rRNA 18S, 28S and of the ribosomal protein S6, a component of the 40S ribosomal subunit.
(TIF)

Table S1 **List of primer sequences and PCR conditions.**
(DOC)

Table S2 **List of primer sequences used for Real Time PCR in a Microfluidic Dynamic Array (Fluidigm® Real-Time PCR).**
(DOC)
