# Supplementary figures and images for: Beta-Catenin/HuR Post-Transcriptional Machinery Governs Cancer Stem Cell Features in Response to Hypoxia
Source: PLoS One. 2013 Nov 15;8(11):e80742. doi: 10.1371/journal.pone.0080742 (PMC3829939; doi:10.1371/journal.pone.0080742)

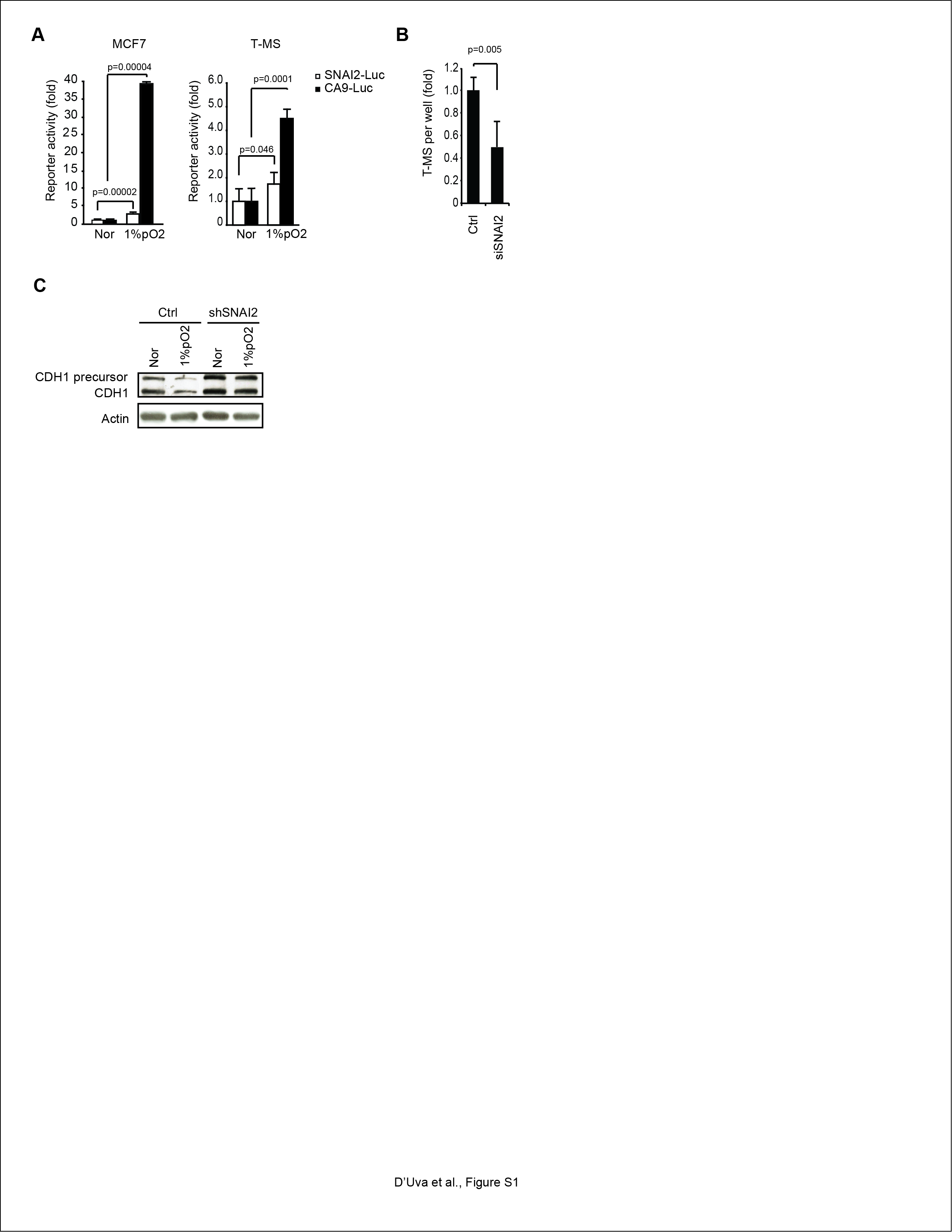

Supplement: Figure S1 — Hypoxia reduces proliferation and differentiation in MCF7 cells, and elicits Snai2 dependent stem cell features in MCF7 cells and T-MS. A, SNAI2 and CA9 promoter luciferase reporter (SNAI2-Luc and CA9-Luc) assay in Nor/1%pO2 MCF7 cells and T-MS; B, T-MS formation upon ctrl/SNAI2-specific siRNA(siSNAI2) transfection of breast cancer primary cells exposed to 1%pO2; C, WB analysis of e-cadherin protein level in SNAI2-specific shRNA retroviral vector (shSNAI2) MCF7 cells upon Nor/1%pO2 conditions. (TIF) [file pone.0080742.s001.tif]

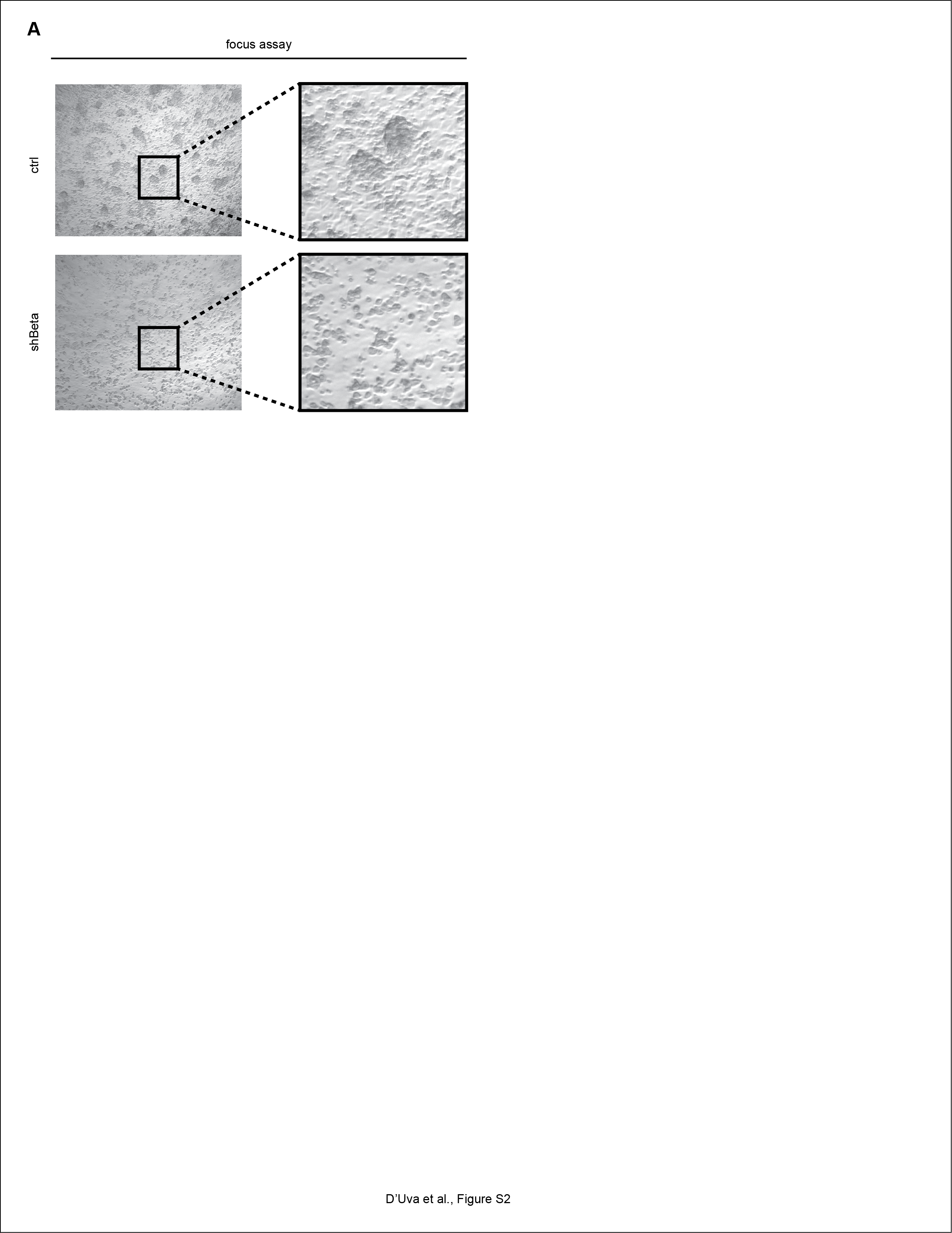

Supplement: Figure S2 — beta-catenin knockdown reduces focus forming capability in hypoxia-exposed MCF7 cells. A, Focus assay in 1%pO2 long-term-exposed (2 weeks) ctrl/shBeta MCF7 cells. (TIF) [file pone.0080742.s002.tif]

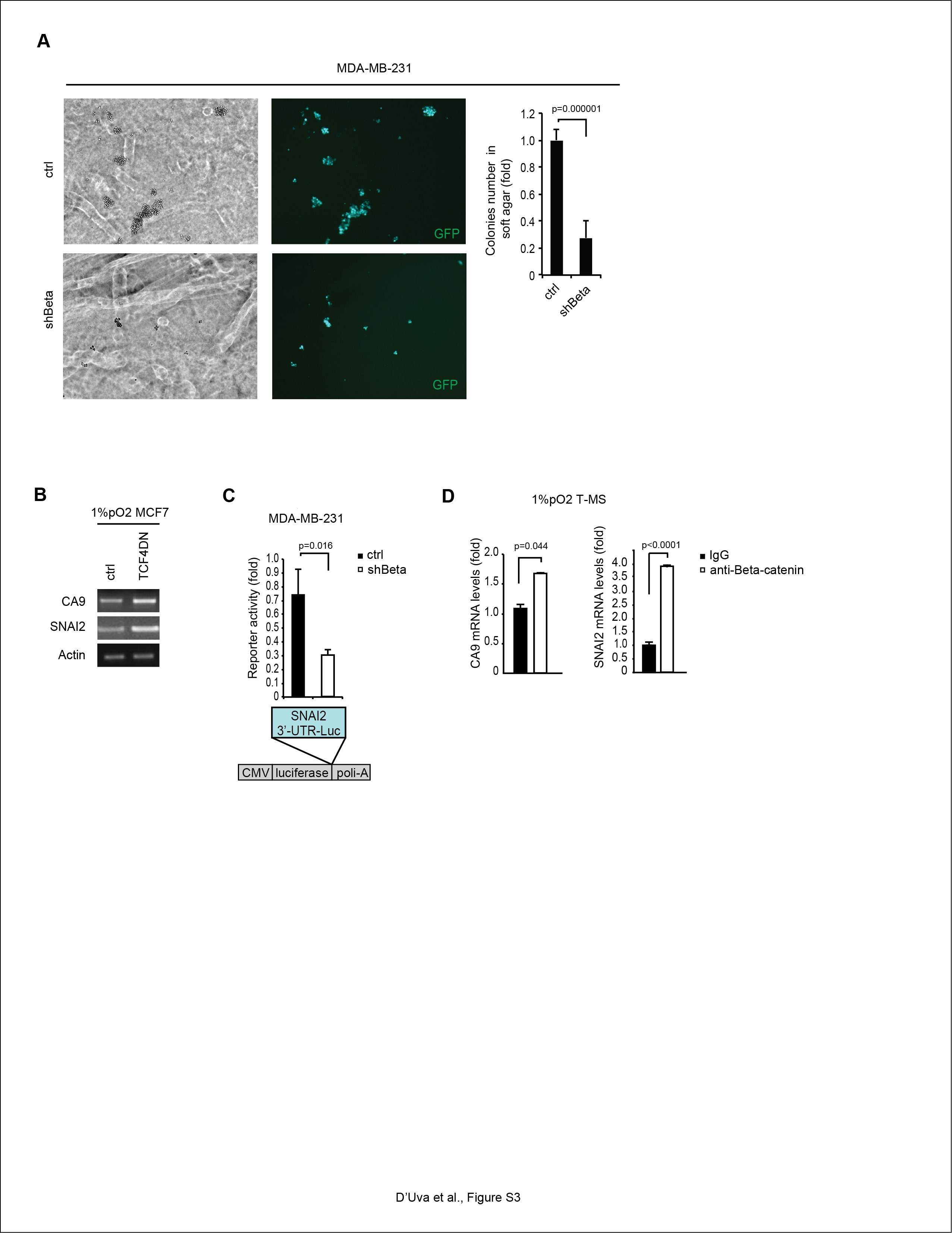

Supplement: Figure S3 — beta-catenin role in breast cancer cell proliferation, colony-forming ability in soft agar and in CA9 and Snai2 transcriptional and post-transcriptional regulation. A, soft agar assay in ctrl/shBeta MDA-MB-231 cells; B, RT-PCR analysis of CA9 and SNAI2 mRNA level in 1%pO2-exposed MCF7 cells transfected with ctrl/TCF4DN; C, SNAI2-3’UTR-Luc assay in ctrl/shBeta MDA-MB-231 cells; D, quantitative CA9 and SNAI2 mRNA immunoprecipitation assay by control IgG/anti-beta-catenin antibody. (TIF) [file pone.0080742.s003.tif]

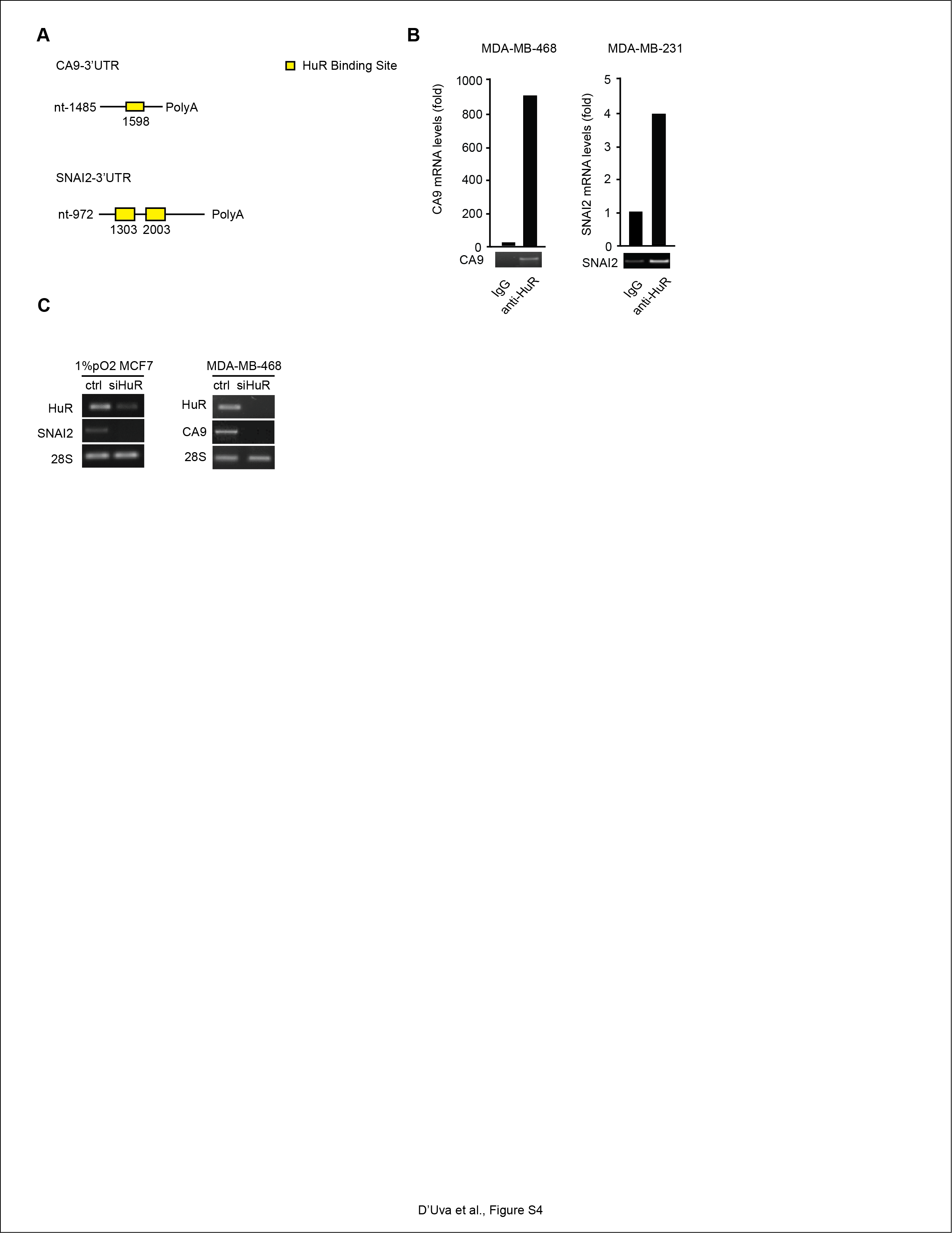

Supplement: Figure S4 — HuR binds and stabilizes CA9 and SNAI2 mRNAs in hypoxic luminal and normoxic basal-like breast cancer cells. A, schematic representation of CA9 and SNAI2 mRNA 3’-UTRs HuR binding sites as predicted by bio-informatics analysis; B, quantitative CA9 and SNAI2 mRNA immunoprecipitation assay by control IgG/beta-catenin antibody; C, Real Time PCR analysis of SNAI2 and CA9 mRNA levels in Ctrl siHuR-transfected/1%pO2-exposed MCF7 cells and normoxic MDA-MB-468 cells. (TIF) [file pone.0080742.s004.tif]

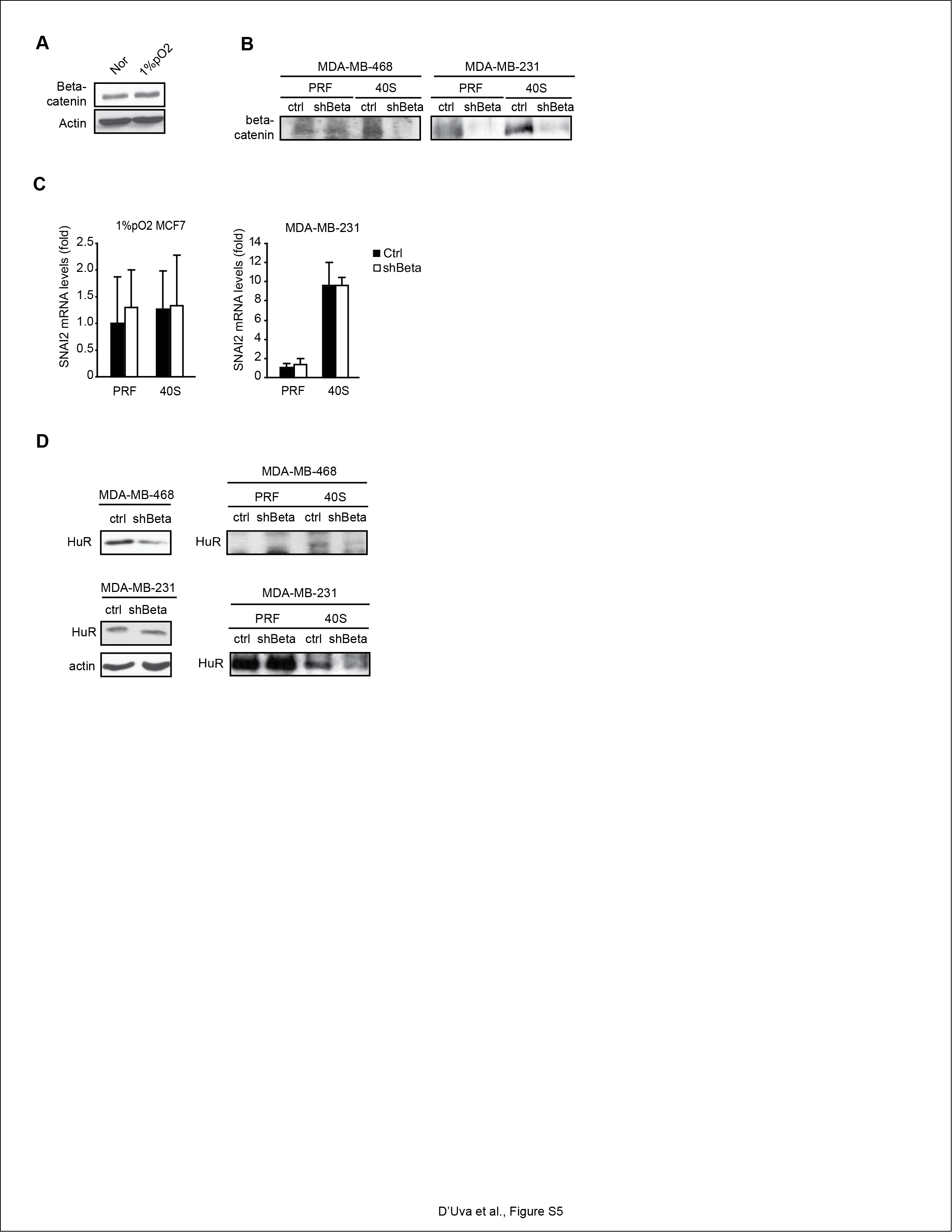

Supplement: Figure S5 — beta-catenin knock-down reduces HuR expression and localization to the ribosomal compartment. A, WB analysis of beta-catenin protein levels in MCF7 cells exposed to 1%pO2; B, WB analysis of beta- catenin protein levels in PRF and 40S cytoplasmic fractions of ctrl/shBeta MDA-MB-468 and MDA-MB-231 cells; C, Real Time PCR analysis of SNAI2 mRNA levels in PRF/40S cytoplasmic fractions of ctrl/shBeta 1%pO2 MCF7 and MDA-MB-231 cells; D, WB analysis of HuR protein levels in total cell lysates and in PRF/40S cytoplasmic fractions of ctrl/shBeta MDA-MB-468 and MDA-MB-231 cells; note that actin protein levels of ctrl/shBeta MDA-MB-468 cells refers to Figure 5D. (TIF) [file pone.0080742.s005.tif]

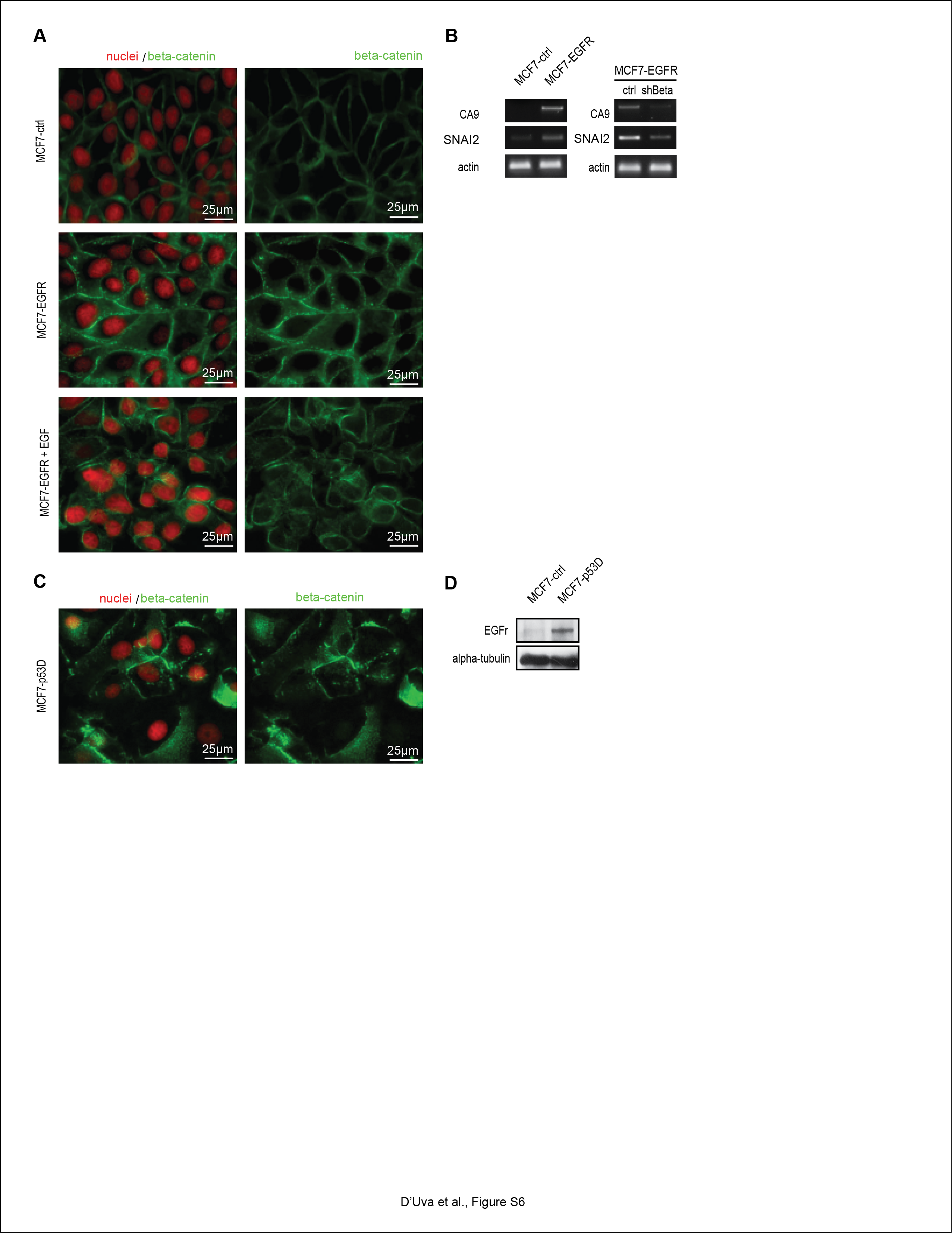

Supplement: Figure S6 — EGFr overexpression and activation promotes the cytoplasmic localization of beta-catenin and the beta-catenin dependent increase in SNAI2 and CA9 mRNA expression. A, IF analysis of beta-catenin in MCF7 cells stably-transfected with empty (MCF7-ctrl) or wild-type EGFR (MCF7-EGFr) vector, in presence/absence of EGF (10ng/ml; 24h); B, RT-PCR analysis of CA9 and SNAI2 mRNA expression levels in MCF7-ctrl/MCF7-EGFR and in MCF7-EGFR cells, transiently transduced with ctrl/shBeta encoding vectors; C, IF analysis of beta-catenin in p53-dominant-negative (p53D) stably transfected MCF7 cells; note that IF of MCF7-ctrl cells refers to panel A; D, WB analysis of EGFR protein levels in MCF7-ctrl/MCF7-p53D. (TIF) [file pone.0080742.s006.tif]

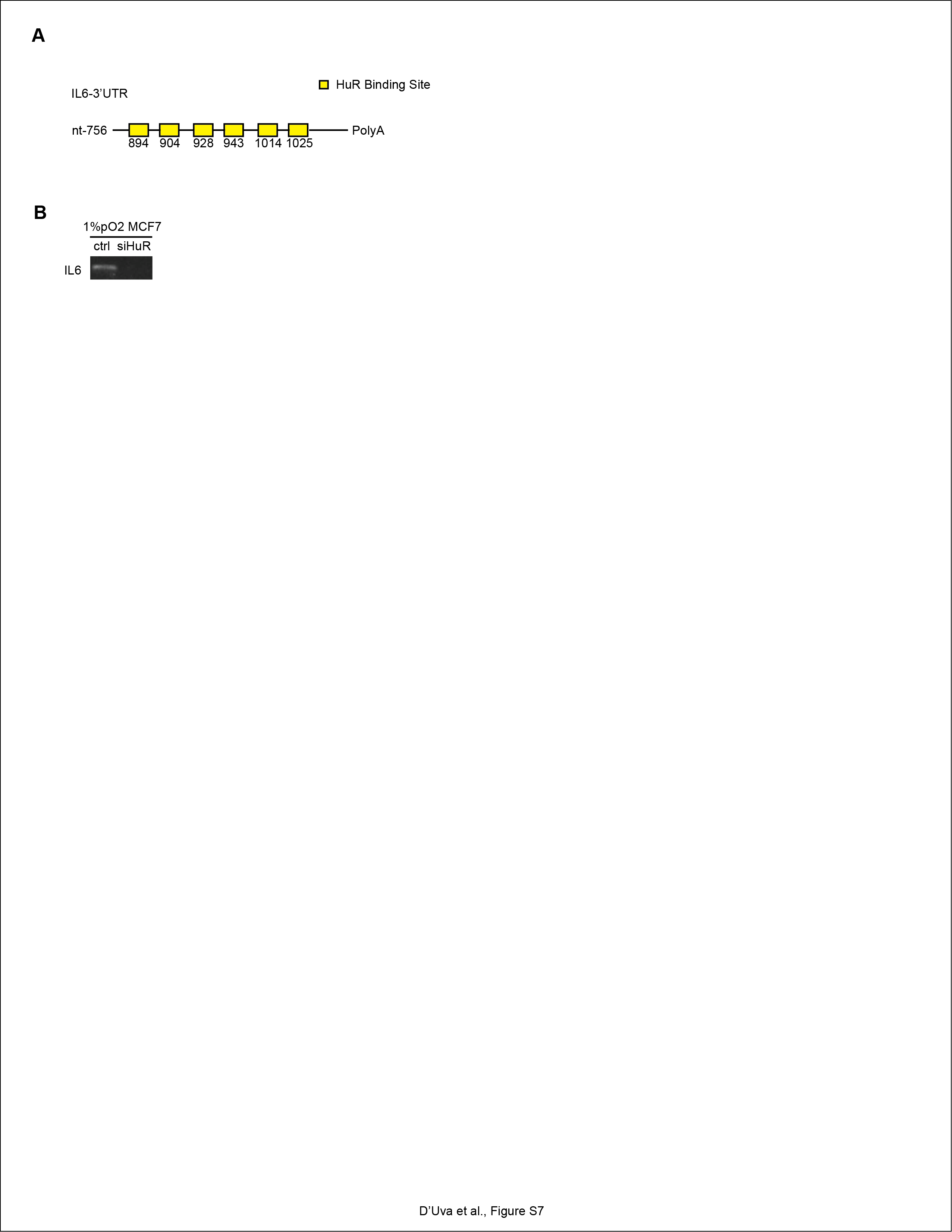

Supplement: Figure S7 — HuR binds and stabilizes IL6 mRNA. A, schematic representation of IL6 3’-UTR HuR binding sites as predicted by bioinformatics analysis; B, RT-PCR analysis of IL6 mRNA levels in 1%pO2 MCF7 cells, transiently transfected with Ctrl/siHuR; note that the loading control (28S ribosomal subunit mRNA) of ctrl/siHuR 1%pO2 MCF7 cells refers to Figure S4C. (TIF) [file pone.0080742.s007.tif]

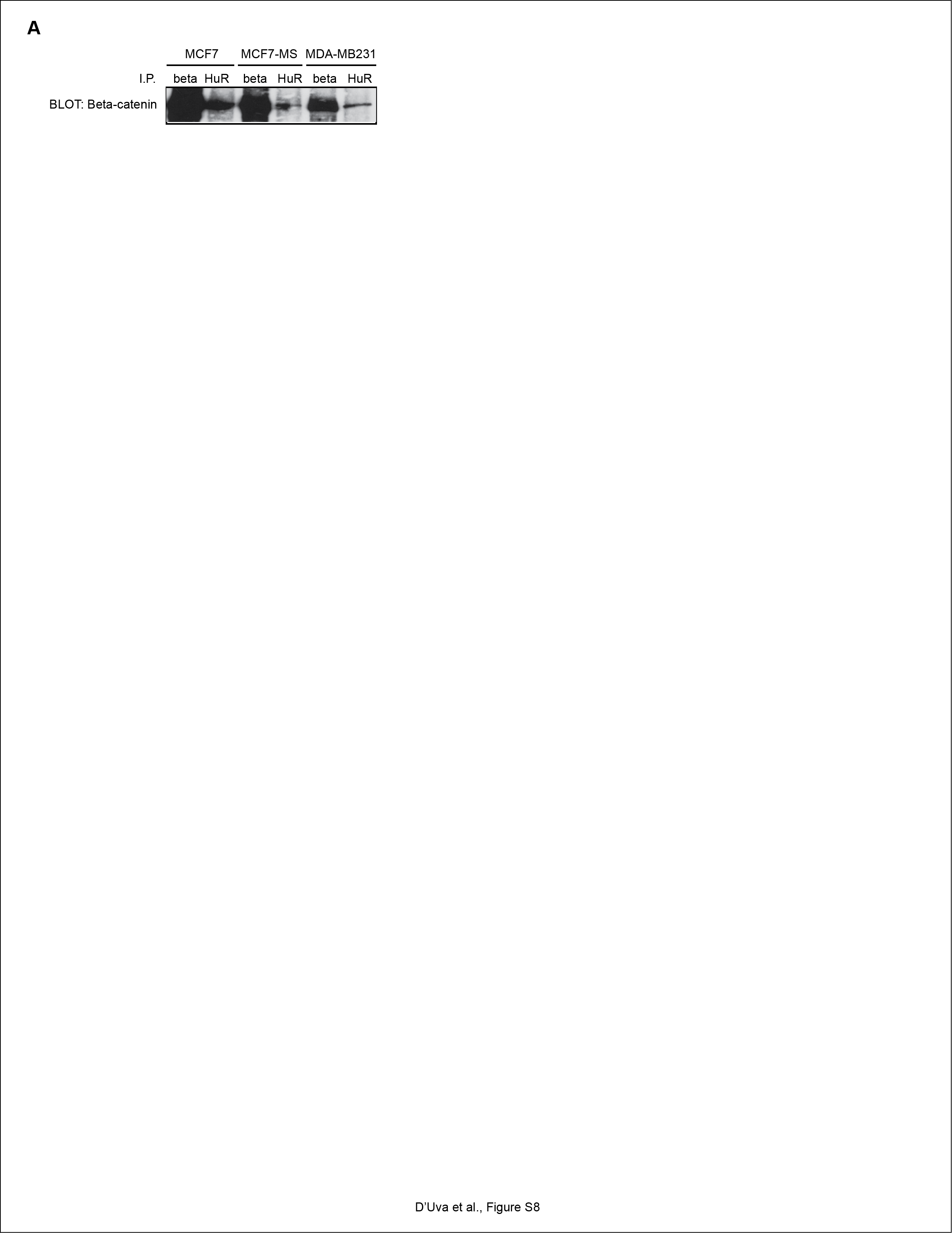

Supplement: Figure S8 — Beta-catenin/HuR physically interacts. A, Co-immunoprecipitation assay of beta-catenin and HuR proteins in MCF7, MCF7-MS and MDA-MB-231 cells. (TIF) [file pone.0080742.s008.tif]

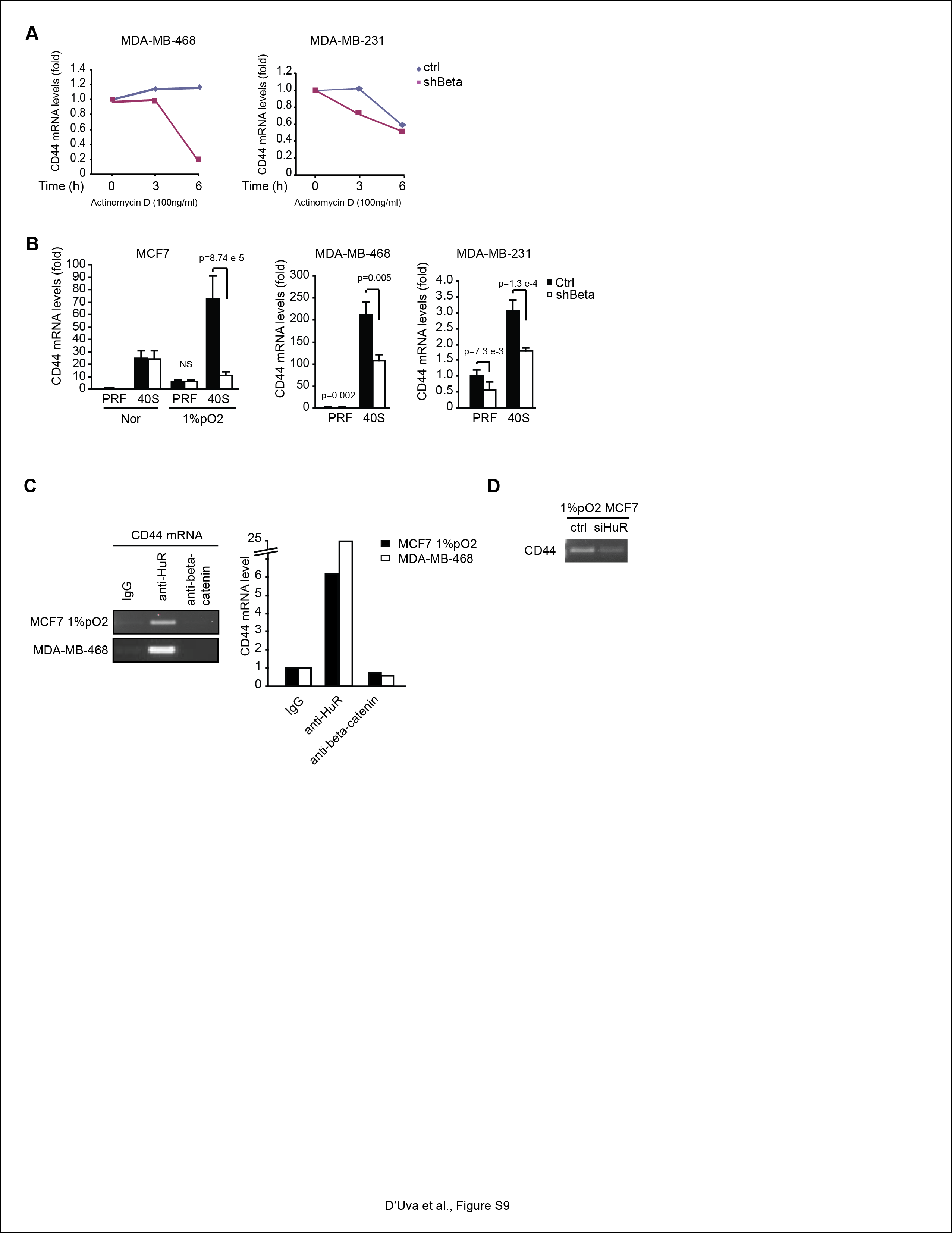

Supplement: Figure S9 — Beta-catenin/HuR post-transcriptional machinery stabilizes CD44 mRNA. A, CD44 mRNA stability assay (actinomycin D, 100ng/ml) in ctrl/shBeta MDA-MB-468 and MDA-MB-231 cells; B, Real Time PCR analysis of CD44 mRNA levels in PRF/40S cytoplasmic fractions of ctrl/shBeta 1%pO2 MCF7, MDA-MB-468 and MDA-MB-231 cells; C, quantitative CD44 mRNA immunoprecipitation assay with control IgG/anti-HuR/anti-beta-catenin antibody in 1%pO2 MCF7 cells and MDA-MB-468 cells; D, RT-PCR analysis of CD44 mRNA levels in scr/siHuR transfected MCF7 cells, exposed to 1%pO2; note that the loading control (28S ribosomal subunit mRNA) of ctrl/shBeta 1%pO2 MCF7 cells refers to Figure S4C. (TIF) [file pone.0080742.s009.tif]

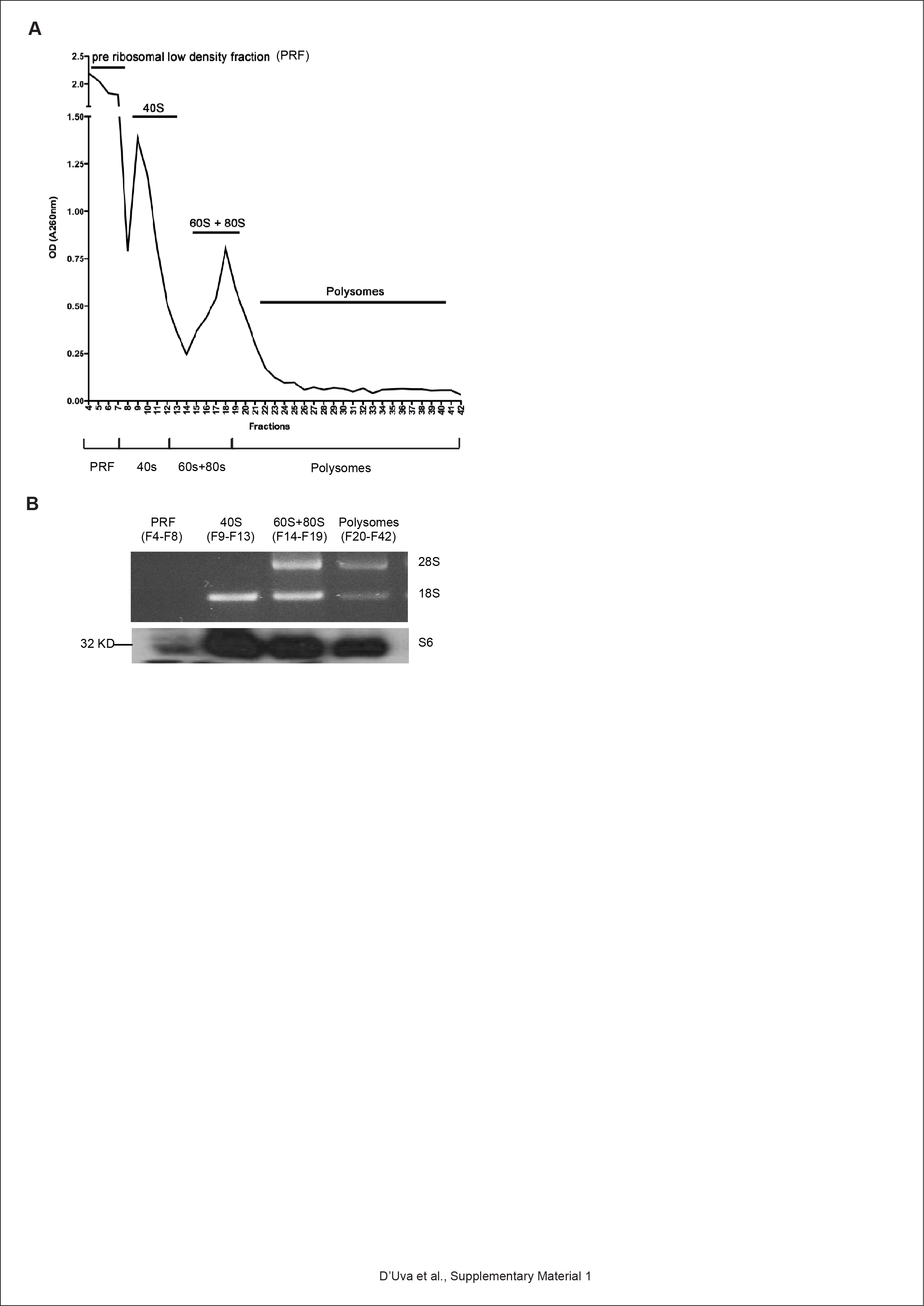

Supplement: Material S1 — Cytoplasmic pre-ribosomal and ribosome fractionation. A, profile of cytoplasmic fractions obtained after centrifugation of cytoplasmic lysates; B, fractions corresponding to low density pre-ribosomal cytoplasm (PRF), 40S, 60-80S and polysomes were pooled and examined in 1% agarose gel and western blot to verify the presence of the rRNA 18S, 28S and of the ribosomal protein S6, a component of the 40S ribosomal subunit. (TIF) [file pone.0080742.s010.tif]
