# Supplementary material for: Beta-Catenin/HuR Post-Transcriptional Machinery Governs Cancer Stem Cell Features in Response to Hypoxia
Source: PLoS One. 2013 Nov 15;8(11):e80742. doi: 10.1371/journal.pone.0080742 (PMC3829939; doi:10.1371/journal.pone.0080742)
Supplement: Table S1 — List of primer sequences and PCR conditions. (DOC) [file pone.0080742.s011.doc]

Table S1 – List of primer sequences and PCR conditions

| **Gene** | **Forward Primer(5’-3’)** | **Reverse primer (5’-3’)** | **Product size [bp]** | **Annealing**  **Temperature**  **[**°C**]** |
| --- | --- | --- | --- | --- |
| CA9 | CAGGGACAAAGAAGGGGATGAC | TTGGAAGTAGCGGCTGAAGTCA | 589 | 61 |
| SNAI2 | AGATGCATATTCGGACCCAC | CCTCATGTTTGTGCAGGAGA | 258 | 60,5 |
| CD44 | GCCAAGAGGGATGCCAAGATGA | CAGCAACCCTACTGATGATGACG | 323 | 62 |
| IL6 | GAGAAAGGAGAACATGTAACAAGAGT | GCGCAGAATGAGATGAGTTGT | 389 | 57 |
| 28S | AACGAGATTCCCACTGTCCC | CTTCACCGTGCCAGACTAGAG | 120 | 60 |
| actin | GGCATCCACGAAACTACCTTCAAC | GACAGGATGCAGAAGGAGATCACT | 168 | 62 |
