# Supplementary material for: Beta-Catenin/HuR Post-Transcriptional Machinery Governs Cancer Stem Cell Features in Response to Hypoxia
Source: PLoS One. 2013 Nov 15;8(11):e80742. doi: 10.1371/journal.pone.0080742 (PMC3829939; doi:10.1371/journal.pone.0080742)
Supplement: Table S2 — List of primer sequences used for Real Time PCR in a Microfluidic Dynamic Array (Fluidigm® Real-Time PCR). (DOC) [file pone.0080742.s012.doc]

Table S2 - List of primer sequences used for Real Time PCR in a Microfluidic Dynamic Array (Fluidigm® Real-Time PCR)

| **Gene** | **Primer name** | **Primer sequence** |
| --- | --- | --- |
| AMIGO | AMIGO_mat | ATACTGCAGCAGGGCAGAAC |
|  | AMIGO_pre | TTTCTGCTTTTTACTCCCTCTGAAT |
|  | AMIGO_universal | GAGTCAGATTTCCCCCTCGT |
| BHLHE40 | BHLHE40_mat | AGACGTGACCGGATTAACGA |
|  | BHLHE40_Pre | CCCAAAGGTGGGACTTCTCT |
|  | BHLHE40_universal | CAAGAACCACTGCTTTTTCCA |
| CXCL1 | CXCL14_mat | CGCACTGCGAGGAGAAGAT |
|  | CXCL14_pre | ACCTCATCCTGCTCCGTTTC |
|  | CXCL14_universal | TTCCAGGCGTTGTACCACTT |
| CXCL14 | CXCL1_mat | ATCCTGCATCCCCCATAGTT |
|  | CXCL1_pre | GAGCAGGGCAGGAGAAGAGT |
|  | CXCL1_universal | CTTCAGGAACAGCCACCAGT |
| DUSP1 | DUSP1_mat | ACTTCATAGACTCCATCAAGAA |
|  | DUSP1_pre | GAAGGGTGTTTGTCCACTGC |
|  | DUSP1_universal | CTCGTCCAGCTTGACTCGAT |
| DUSP4 | DUSP4_mat | CCACAGAGCCCTTGGACCT |
|  | DUSP4_pre | CCTGTGCCAAGCACTTTACC |
|  | DUSP4_universal | GAGGAAGGGAAGGATCTCCA |
| ENC1 | ENC1_mat | TTTGTCAGCACCTGGAAACA |
|  | ENC1_pre | CATCACACAAATCCTTCATGCT |
|  | ENC1_universal | AGACTTGGCCTCTCCGAAGT |
| EOMES | EOMES_mat | CGCCACCAAACTGAGATGAT |
|  | EOMES_pre | GCCTGTTCTAGGACATCCCAATTA |
|  | EOMES_universal | TTGTAGTGGGCAGTGGGATT |
| EREG | EREG_mat | TCCATCTTCTACAGGCAGTCCT |
|  | EREG_pre | CTTCCATGAAGGCTGCAGAA |
|  | EREG_universal | AGCCACACGTGGATTGTCTT |
| ERRIF1 | ERRIF1(5' side)_pre_FW | TCCTAATGGAGGTATTTCTGAATTGT |
|  | ERRIF1(5' side)_pre_REV | CTGGGACATCTCCAAACCTG |
|  | ERRIF1(5' side)_mat_REV | CCTCTTCATGTGGTCCCAAG |
|  | ERRIF1(5' side)_mat_FW | GCCACTGCTTTGCAGAAAAT |
| FGD4 | FGD4_mat | AGCTGCTCGGAACACTTCAG |
|  | FGD4_pre | ACCTGATCAGTTTCCCCTATTTCT |
|  | FGD4_universal | TGGGCACACAGTACAGCAAC |
| FLI1 | FLI1_mat | TCCCTCCTCATGTCATCTCC |
|  | FLI1_pre | CACGGAAGTGCTGTTGTCAC |
|  | FLI1_universal | TCGGTGTGGGAGGTTGTATT |
| FOXO3 | FOXO3_mat | CTTCAAGGATAAGGGCGACA |
|  | FOXO3_pre | CTCGGTTTTGGACCATTCTG |
|  | FOXO3_universal | TCTTGCCAGTTCCCTCATTC |
| GDF15 | GDF15_mat | GAGCTGGGAAGATTCGAACA |
|  | GDF15_pre | GTTCCTGGAAAACGGTAGGC |
|  | GDF15_universal | CGAGAGATACGCAGGTGCAG |
| GFPT2 | GFPT2_mat | CCTGTGCCAAGTGTGTGAGA |
|  | GFPT2_pre | CGGCTGGAGTACAGAGGCTA |
|  | GFPT2_universal | GACTTCGTGATTATTCCCATCG |
| HBEGF | HBEGF_mat | GCTGTGGTGCTGTCATCTGT |
|  | HBEGF_pre | CTTTGGAAGGACCTGCTCTG |
|  | HBEGF_universal | TCATGCCCAACTTCACTTTCT |
| IL8 | IL8_mat | CGGAAGGAACCATCTCACTG |
|  | IL8_pre | AAAGGAAGTAGCTGGCAGAGC |
|  | IL8_universal | AGCACTCCTTGGCAAAACTG |
| HES1 | HES1_mat | AAGGCGGACATTCTGGAAAT |
|  | HES1_pre | TGACCCGTCTGTCTCTTTCTG |
|  | HES1_universal | TACTTCCCCAGCACACTTGG |
| IER3 | IER3_mat_FW | GGACTACGCTCTGGACCTCA |
|  | IER3_mat_REV | AGTGCGGGGAGTCACAGTTA |
|  | IER3_pre_FW | CGACCTGACCTGTCTCCTGT |
|  | IER3_pre_REV | GCAGAAAGAGAAGCCTTTTGG |
| IL6 | IL6_mat | GCCAGAGCTGTGCAGATGAG |
|  | IL6_pre | CATCATCCCATAGCCCAGAG |
|  | IL6_universal | TCAGGGGTGGTTATTGCATC |
| IL1R1 | IL1R1_mat_FW | TCATAGCTCTACTGATTTCTTCTCTGG |
|  | IL1R1_mat_REV | CGAACATCAATTTCATTTGCAG |
|  | IL1R1_pre_FW | ATTGCTTCCACCCTTCTTCC |
|  | IL1R1_pre_REV | AGGACAGGGACGAACATCAA |
| LOX | LOX_mat | CGCTGTGACATTCGCTACAC |
|  | LOX_pre | AAAGGTTGACTTTAAATTTGTCTGTTG |
|  | LOX_universal | CCATTGGGAGTTTTGCTTTG |
| MAOA | MAOA_mat | TCTGACCAATTTTTCTCTTTTTGC |
|  | MAOA_pre | GGACAGGGTTGGAGGAAGAA |
|  | MAOA_universal | TGCCCAGCTCCTTAGACAAG |
| NEXN | NEXN_mat | CCGAAAGAAGCAAGCTGAAG |
|  | NEXN_pre | TGGCTAATTCTGTGCCTTTTG |
|  | NEXN_universal | TGCTGTGTCTTGGTTTTCCTC |
| NRG1 | NRG1_mat | TGGTTCAAGAATGGGAATGAA |
|  | NRG1_pre | TGACACCACTTTGGTCCTGA |
|  | NRG1_universal | CTCTCCAGAATCAGCCAGTGA |
| PIK3R1 | PIK3R1_mat | TGTTGCACCAGGTTCTTCG |
|  | PIK3R1_pre | GGTGGGATTTTGTTGTTTGC |
|  | PIK3R1_universal | GGCAAACTGCTCTGCAAGAT |
| SCNN1B | SCNN1B_mat | CTCCGTAGGCTTCAAGACCAT |
|  | SCNN1B_pre | CATTCCTTCCCCCTAACCAG |
|  | SCNN1B_universal | TCTCCAGGACAGCTTCCATC |
| SEMA6A | SEMA6A_mat | AACACTGGCAATGTCAAGCA |
|  | SEMA6A_pre | TCAACACAGCTAGGGCATGA |
|  | SEMA6A_universal | TTGTCCTGGCAACGTTTTCT |
| SERPINB2 | SERPINB2_pre | TTTGATGGCTACTCAGAAGATTCA |
|  | SERPINB2_mat | TGGGTCAAGACTCAAACCAAA |
|  | SERPINB2_universal | TGGTATCCCCATCTACAGAACC |
| SLC2A3_chr12 | SLC2A3_chr12_mat | TGAGGACAGAGGAGAAGTTAGGAG |
|  | SLC2A3_chr12_pre | CCTTTTCCGTCGGACTCTTC |
|  | SLC2A3_chr12_universal | TAAAGCAGCCACCAGTGACA |
| SPRY4 | SPRY4_mat | GGCGTCTGCGAGTACAGC |
|  | SPRY4_pre | GGATTAGGCATCCTGCTCAA |
|  | SPRY4_universal | CTGAGCATCAGGCTGCAAAC |
| VEGFA | VEGFA_mat | AGGAGGAGGGCAGAATCATC |
|  | VEGFA_pre | GCATTACAGAGCTGGGTGGA |
|  | VEGFA_universal | AGCTGCGCTGATAGACATCC |
| TNFAIP3 | TNFAIP3_mat | ACCCTGGAAAGCCAGAAGAA |
|  | TNFAIP3_pre | TGCTGGGTCTTACATGCAGAT |
|  | TNFAIP3_universal | CTGAACGCCCCACATGTACT |
| TGFA | TGFA_2_pre | CCCTGGAGAGCTAGGGTAACA |
|  | TGFA_2_mat | GTTTTTGGTGCAGGAGGACA |
|  | TGFA_2_universal | CACCAACGTACCCAGAATGG |
| TBP | TBP__F | CTTCACACGCCAAGAAACAGT |
|  | TBP__R | GCTGGCCCATAGTGATCTTT |
| GAPDH | GAPDH_FW | ACCCACTCCTCCACCTTTGA |
|  | GAPDH_REV | CTGTTGCTGTAGCCAAATTCGT |
| B2M | B2M_FW | GGCATTCCTGAAGCTGAC |
|  | B2M_REV | TCTTTGGAGTACGCTGGATAG |
